# In-depth assessment of BRAF, NRAS, KRAS, EGFR, and PIK3CA mutations on cell-free DNA in the blood of melanoma patients receiving immune checkpoint inhibition

**DOI:** 10.1186/s13046-025-03457-w

**Published:** 2025-07-12

**Authors:** Isabel Heidrich, Charlotte Rautmann, Cedric Ly, Robin Khatri, Julian Kött, Glenn Geidel, Alessandra Rünger, Antje Andreas, Inga Hansen-Abeck, Finn Abeck, Anne Menz, Stefan Bonn, Stefan W. Schneider, Daniel J. Smit, Christoffer Gebhardt, Klaus Pantel

**Affiliations:** 1 Institute of Tumor Biology, Fleur Hiege Center for Skin Cancer Research, University Medical Center Hamburg-Eppendorf, Hamburg, Germany; 2 Department of Dermatology and Venereology, Fleur Hiege Center for Skin Cancer Research, University Medical Center Hamburg-Eppendorf, Hamburg, Germany; 3 Mildred Scheel Cancer Career Center HaTriCS4, University Medical Center Hamburg-Eppendorf, Hamburg, Germany; 4 Institute of Systems Immunology, University Medical Center Hamburg-Eppendorf, Hamburg, Germany; 5 Institute for Medical Systems Bioinformatics, Center for Molecular Neurobiology (ZMBH), University Medical Center Hamburg-Eppendorf, Hamburg, Germany; 6 Hamburg Center for Translational Immunology (HCTI), University Medical Center Hamburg-Eppendorf, Hamburg, Germany; 7 Center for Biomedical AI, University Medical Center Hamburg-Eppendorf, Hamburg, Germany; 8 Institute of Pathology, University Medical Center Hamburg-Eppendorf, Martinistrasse, Hamburg, Germany; 9 Department of Tumor Biology, University Medical Center Hamburg-Eppendorf, Martinistraße 52, Hamburg, 20246 Germany

**Keywords:** CtDNA, Liquid Biopsy, Melanoma, BRAF, NRAS, KRAS, EGFR, PIK3CA

## Abstract

**Introduction:**

Circulating tumor DNA (ctDNA) holds promise for guiding immune checkpoint inhibitor (ICI) therapy and stratifying responders from non-responders. While tumor-informed ctDNA detection approaches are sensitive and mutation-inclusive, they require tumor tissue, which limits applicability in real-world settings. Conversely, tumor-agnostic methods often have limited genomic coverage. In this study, we evaluated a tumor-agnostic, broad-panel ctDNA assay in patients with advanced melanoma treated with ICI.

**Methods:**

We conducted a prospective analysis of 241 longitudinal samples from 39 patients with unresectable stage III/IV melanoma using a SYSMEX targeted NGS panel covering 1,114 COSMIC mutations. Plasma samples were collected at baseline and during ICI therapy. The assay’s sensitivity reached seven mutant molecules, corresponding to a 0.07% mutation allele frequency (MAF). ctDNA profiles were compared with matched tumor tissue and correlated with clinical features and survival.

**Results:**

At baseline, ctDNA was detected in 64.5% of patients. Common mutations included *BRAF*^V600E^ (43.8%) and *NRAS*^G12D^ (36.4%), followed by *KRAS, EGFR,* and *PIK3CA* variants. Overall tissue–plasma concordance was 51.6%, with more extended biopsy–plasma intervals associated with discordance (*p* = 0.0105). Notably, 12.2% of cases exhibited partial concordance, characterized by shared mutations and additional plasma-only alterations, underscoring the complementary value of blood-based profiling. Persistent or re-emerging ctDNA positivity post-therapy correlated with shorter progression-free survival (PFS, *p* = 0.003), while ctDNA-negative patients showed significantly improved outcomes. Patients that remained ctDNA-negative had significantly longer progression-free survival (median not reached) compared to those with persistent ctDNA positivity (median 3 months) or those converting to positive (median 7.5 months; *p* = 0.0073). Early *NRAS* and *KRAS* ctDNA levels strongly predicted poor response (*p* = 0.0069 and *p* = 0.028). The prognostic impact extended beyond canonical drivers, as non-hotspot variants also correlated with the outcome. Notably, even low-level ctDNA persistence (5–10 MM/mL) carried adverse prognostic implications (*p* = 0.0054). Concerning a shorter PFS, ctDNA positivity was also associated with elevated S100 levels (*p* = 0.047). Organ-specific mutation enrichment (e.g., KRAS^G12D^ in brain, EGFR^G719A^ in lymph nodes) suggested possible metastatic tropism.

**Conclusion:**

Broad tumor-agnostic ctDNA analysis effectively identified clinically relevant mutations and predicted outcomes in ICI-treated melanoma patients. This approach enables tissue-independent and real-time ctDNA monitoring and may inform patient selection and therapeutic strategies in future interventional trials.

**Supplementary Information:**

The online version contains supplementary material available at https://doi.org/10.1186/s13046-025-03457-w.

## Background

Melanoma is the most aggressive skin cancer, and incidence is increasing in Europe and the US. In addition to targeted therapies, immunotherapy has significantly altered the therapeutic landscape for melanoma patients; however, resistance to treatment remains a significant concern [1–3]. Therefore, biomarkers that can predict the clinical outcome of melanoma patients receiving immunotherapy are warranted [4]. Over the past decade, circulating tumor DNA (ctDNA) has emerged as a promising Liquid Biopsy (LB) biomarker, enabling real-time risk assessment, monitoring of tumor burden, and therapeutic response in melanoma patients undergoing immunotherapy [5–8].

However, various technologies are available for analyzing ctDNA in blood from melanoma patients, and it remains unclear which technology is best suited for use in a real-world clinical setting. In principle, tumor-agnostic or tumor-informed assays can be used for ctDNA analysis. Tumor-informed assays require sequencing (WES or WGS) of the primary tumor to obtain patient-specific probes for plasma analysis [6, 9, 10]. However, primary melanomas are often resected on an outpatient basis, which, together with the fact that the amount of tissue, especially in metastasis, is generally minimal, may limit the use of tumor-based ctDNA testing in a real-world setting. Tumor-agnostic tests target a pre-selected panel of genes relevant to melanoma (or tumor entities) and do not require access to tumor tissue [11]. PCR-based technologies, such as digital droplet PCR (ddPCR), can also be highly sensitive; however, the number of pre-selected mutations is limited, which can lead to false-negative results if tumors evolve during progression to carry rarer variants not covered by the ddPCR panel [12].

Here, we combined the advantages of targeted PCR analysis with a more comprehensive Next Generation Sequencing (NGS) analysis including 1,114 cosmic mutations testing five cancer genes relevant in melanoma biology (*BRAF, NRAS, KRAS, EGFR*, and *PIK3CA*) on cell-free DNA from blood plasma samples obtained from melanoma patients receiving gold standard immunotherapy (anti-CTLA4-Antibody, anti-PD1-Antibody). Our study provides insights into the prevalence and dynamics of known and novel melanoma-associated mutations in ctDNA and their association with clinical parameters, treatment response, and survival outcomes.

## Materials and methods

### Patient cohort

This retrospective study included 39 patients with advanced melanoma who received ICI (combination of CTLA-4 inhibitor Ipilimumab, (YERVOY®, Bristol-Myers Squibb) 3 mg/kg body weight and PD-1 inhibitor Nivolumab (Opdivo®, Bristol-Myers Squibb) 1 mg/kg body weight every 3 weeks or Nivolumab (Opdivo®, Bristol-Myers Squibb) monotherapy 480 mg every 4 weeks; PD-1 inhibitor Pembrolizumab monotherapy 200 mg every 3 weeks or 400 mg every 6 weeks (KEYTRUDA**®,** Merck & Co, MSD)) at the University Skin Cancer Center Hamburg, Germany. Patients were enrolled between March 2018 and February 2020. All patients provided informed consent to participate in this study, which was approved by the Ethics Committee of the Hamburg Medical Association (PV5392). Inclusion criteria were: (i) a confirmed diagnosis of unresectable stage III or stage IV according to AJCC cutaneous or mucosal melanoma staging and classification (8th edition), (ii) at least 18 years of age, and (iii) at least 6 months of clinical follow-up.

### ctDNA analysis

Whole blood samples were centrifuged twice (1. Cycle: 300 x g, 10 min, 23 °C, 2. Cycle: 1800 x g, 10 min, 23 °C), and plasma was stored at −80 ◦C until cell-free DNA (cfDNA) extraction. cfDNA was extracted using the QIA-amp Circulating Nucleic Acid Kit (Qiagen, Valencia, CA, USA, Cat No. 55114) according to the manufacturer’s instructions. cfDNA was quantified using a Qubit™ 4 Fluorometer (Invitrogen™, Cat. No. Q33238, Carlsbad CA,2008, USA) with the Qubit™ dsDNA HS Assay Kit (Invitrogen™, Cat. No. 32854). cfDNA libraries were generated using the beta version of the Plasma-SeqSensei™ SOLID CANCER RUO kit (Sysmex Inostics GmbH). The libraries were then quantified on a Bioanalyzer (Agilent, Santa Clara, CA, USA) and sequenced on a NextSeq 550 instrument (Illumina, San Diego, CA, USA). The sequencing data were analyzed using proprietary software packages developed by Sysmex Inostics. Circulating tumor DNA (ctDNA) analysis was performed using the Plasma-SeqSensei™ Solid Cancer IVD kit (Sysmex Inostics), an amplicon-based assay specifically designed for high-sensitivity detection of somatic mutations in plasma-derived cfDNA. The input amount of cfDNA for library preparation ranged between 7.6 ng and 56.6 ng per time point per sample. It was individually determined for each time point based on the availability and quantification of cfDNA. The panel targets clinically relevant regions from established driver genes. Targeted regions were based on Ensembl canonical transcripts (Table 1), with the following Ensembl and RefSeq IDs used to define the coding regions:

**Table 1:** RefSeq IDs of *BRAF, EGFR, KRAS, NRAS,* and *PIK3CA* used to define the coding regions

Gene	Ensembl ID	RefSeq ID
*BRAF*	ENST00000288602.6	NM_004333.6
*EGFR*	ENST00000275493.2	NM_005228.5
*KRAS*	ENST00000256078.4	NM_033360.4
*NRAS*	ENST00000369535.4	NM_002524.5
*PIK3CA*	ENST00000263967.3	NM_006218.4

Library preparation was performed using an amplicon-based enrichment strategy, optimized for fragmented cfDNA. Unique Molecular Identifiers (UMIs) were incorporated into each cfDNA molecule before amplification, allowing for error correction and reliable discrimination between true somatic mutations and PCR or sequencing artifacts. The libraries were then quantified on a Bioanalyzer (Agilent, Santa Clara, CA, USA) and sequenced on a NextSeq 550 instrument (Illumina, San Diego, CA, USA). The sequencing data were analyzed using proprietary software packages developed by Sysmex Inostics.

### Analytical sensitivity and specificity

The analytical performance of the assay was validated by CLSI guidelines (EP17-A2, EP05-A3, EP06-A, and EP07-A2). The limit of detection (LoD95) was determined as 6.21 mutant molecules (95% CI: 5.47–7.26), with a conservative reporting threshold set at ≥ 7 mutant molecules. Analytical specificity was verified through in-silico BLAST analysis to exclude cross-reactivity with the human genome and common microbial DNA. In addition, a position-specific limit of blank (LoB) was established using cfDNA from healthy donors and integrated into the variant calling algorithm to minimize false positives due to background noise.

The analysis pipeline is based on SafeSEQ™ technology, which comprises three main steps: UID Family Grouping: Reads sharing the same UMI are grouped to reduce random sequencing errors. Consensus Building: A consensus sequence is generated for each UID group, enhancing accuracy and reproducibility. Variant Calling: Consensus reads are aligned to the reference genome, and only variants above the defined LoD and outside the LoB are reported. Further technical specifications and validation data are available in the manufacturer’s Instructions for Use (IFU, ZR150537.R4) and via the Caresphere Academy’s Plasma-SeqSensei™ online training platform.

### Analysis of other blood parameters

Lactate dehydrogenase (LDH), S100B, D-dimers, and C-reactive protein (CRP) levels, as well as leukocyte, neutrophil, lymphocyte, and platelet counts, were measured in peripheral blood by routine clinical laboratory analysis using an ADVIA® 2120i System analyzer (Siemens). LDH was measured by spectrophotometry (Atellica Solution CH930), S100 by chemiluminescence (Liaison XL), and CRP by turbidimetry (Atellica Coag 360 and Atellica Solution CH930, respectively). The following counts/measurements were considered normal, according to clinical routine cut-off values: platelets (150–300*10^9^/L), LDH (120–246 U/L), S100 (< 0.150 µg/L), D-dimers (0.21–0.52 mg/L), CRP (< 5 mg/L), lymphocytes (1,400–4,800/µL), neutrophils (1,800–7,000/µL), platelets (150,000–400,000/µL).

### Tissue analysis

Targeted next-generation sequencing (NGS) was conducted to identify mutations in the *PIK3CA*, *NRAS*, *KRAS*, and *BRAF* genes using DNA extracted from formalin-fixed, paraffin-embedded (FFPE) tumor tissue. Library preparation was performed using the QIAseq Targeted DNA Panel (Qiagen), custom NGS Qiagen panels, and the AmpliSeq™ Focus Panel (Thermo Fisher Scientific), depending on sample availability and time of analysis. Sequencing was performed on Illumina platforms according to the manufacturer’s protocols. Bioinformatic processing included alignment to the GRCh37/hg19 human reference genome, variant calling, and annotation using validated in-house pipelines. Variants were filtered based on a minimum read depth of 500 × and a variant allele frequency (VAF) ≥ 5%. All clinically relevant variants were manually reviewed and classified by ACMG/AMP guidelines.

### Statistical analysis

The statistical analysis and visualization were performed using GraphPad Prism (version 10, GraphPad Software, San Diego, CA, USA). For data preprocessing, the Python packages pandas version 1.3.4 and pyvcf version 0.6.8 were used. The heatmap (Fig. 6) was plotted using the Python matplotlib package version 3.5.0. For data analysis and visualization, the following packages were used in RStudio: ggplot2 (version 3.3.4), finalfit (version 1.0.7), survminer (version 0.4.9), survival (version 3.5–7), as well as dplyr (version 1.1.4) and data.table (version 1.17.0) to calculate Z-scores, and pheatmap (version 1.0.12) for data visualization. The Z-scores were computed by standardizing the data (subtracting the mean and dividing by the standard deviation). Laboratory measurements reported as below the lower limit of detection (LoD) were imputed as LoD/2, a commonly used conservative approach to account for left-censored data in biomedical research, ensuring their inclusion in continuous variable analyses. Categorical variables were analyzed using Fisher’s exact test. Continuous variables were first assessed for normal distribution using the Shapiro–Wilk test, and if applicable, equality of variance was tested using the Levene test. Differences in the mean of continuous variables of two groups were analyzed by Student’s t-test (parametric data with equal variance), Welch’s t-test (parametric data with unequal variance), or the Mann–Whitney U test (non-parametric data), where applicable. Survival curves were plotted using the Kaplan–Meier method, and differences in survival times (overall survival (OS) and progression-free survival (PFS)) were analyzed using the log-rank test (Mantel–Cox). A p-value of < 0.05 was considered statistically significant.

## Results

### Patient cohort and distribution of ctDNA mutations

We analyzed plasma-derived ctDNA for the five different cancer-associated genes (*BRAF, EGFR, KRAS, NRAS, PIK3CA)* from 241 timepoints of 39 melanoma patients, of which 89.7% were in metastatic American Joint Committee on Cancer (AJCC) stage IV and 10.3% in unresectable AJCC stage III; 66.7% of these patients were male and 33.3% were female. The enrolled patients had a median age of 71 years (range, 39–87 years). In addition to cutaneous melanoma (*n* = 35), patients also presented with mucosal melanoma (*n* = 4). 64.1% of patients received anti-CTLA-4/anti-PD-1 combination therapy (Ipilimumab + Nivolumab), whereas 28.2% received Pembrolizumab monotherapy and 7.7% received Nivolumab as a monotherapy targeting PD-1. For most of the patients (74.4%) included in this study, first-line treatment was administered.

Overall, 40.5% of patients with available primary tumor tissue had a *BRAF* mutation in their tumor tissue (35.1% *BRAF*^V600E^, 2.7% *BRAF*^V600R^, *BRAF*^V600K^ 2.7%), 18.9% of patients had a *NRAS* mutation (10.8% *NRAS*^Q61R^, 8.1% *NRAS*^Q61K^) and one patient (2.9%) had a *KRAS* mutation *(KRAS*^*G12*^) in their tissue. No *PIK3CA* mutations were found in the tissue (Table 2). Blood samples were collected from 31 patients at the start of treatment (at baseline) and every 3–4 weeks thereafter, and from 8 patients at 12 weeks before and after progression under immunotherapy.

**Table 2:** Demographic, clinical, and pathological parameters of the study population

	Total N		N (%)
Sex	39 (100.0)	Male	26 (66.7)
		Female	13 (33.3)
Age group	39 (100.0)	< 65	12 (30.8)
		≥ 65	27 (69.2)
Primary melanoma site	39 (100.0)	Cutaneous	35 (89.7)
		Mucosal	4 (10.3)
*BRAF* status (tissue)	37^a^ (94.9)	Wildtype	22 (59.5)
		BRAF V600E	13 (35.1)
		BRAF V600K	1 (2.7)
		BRAF V600R	1 (2.7)
*KRAS* status (tissue)	34^a^ (87.2)	Wildtype	33 (97.1)
		Mutated	1 (2.9)
*NRAS* status (tissue)	37^a^ (94.9)	Wildtype	30 (81.1)
		NRAS Q61R	4 (10.8)
		NRAS Q61K	3 (8.1)
*PIK3CA* status (tissue)	36^a^ (92.3)	Wildtype	36 (100.0)
AJCC	39 (100.0)	Stage III	4 (10.3)
		Stage IV	35 (89.7)
T	39 (100.0)	T0	10 (25.6)
		T1	3 (7.7)
		T2	5 (12.8)
		T3	5 (12.8)
		T4	14 (35.9)
		Tx	2 (5.1)
N	39 (100.0)	N0	14 (35.9)
		N1	9 (23.1)
		N2	8 (20.5)
		N3	8 (20.5)
M	39 (100.0)	M0	5 (12.8)
		M1	34 (87.2)
Baseline therapy	39 (100.0)	Ipilimumab + Nivolumab	25 (64.1)
		Pembrolizumab	11 (28.2)
		Nivolumab	3 (7.7)
No. of treatment lines	39 (100.0)	First line	29 (74.4)
		Second line	8 (20.5)
		Third line	2 (5.1)
Immune-related adverse events	37^b^ (94.9)	No	18 (48.6)
		Yes	19 (51.4)
ctDNA at baseline	31 (79.5)	Negative	11 (35.5)
		Positive	20 (64.5)

*T* Tumor, *N *lymph nodes, *M *distant metastasis; according to the 8th edition of the AJCC cancer staging manual. EGFR analysis in tissue was not performed

^a^The patients missing here had not enough tissue for mutational analysis

^b^The missing information on the irAE status was not available

**Fig. 1:**
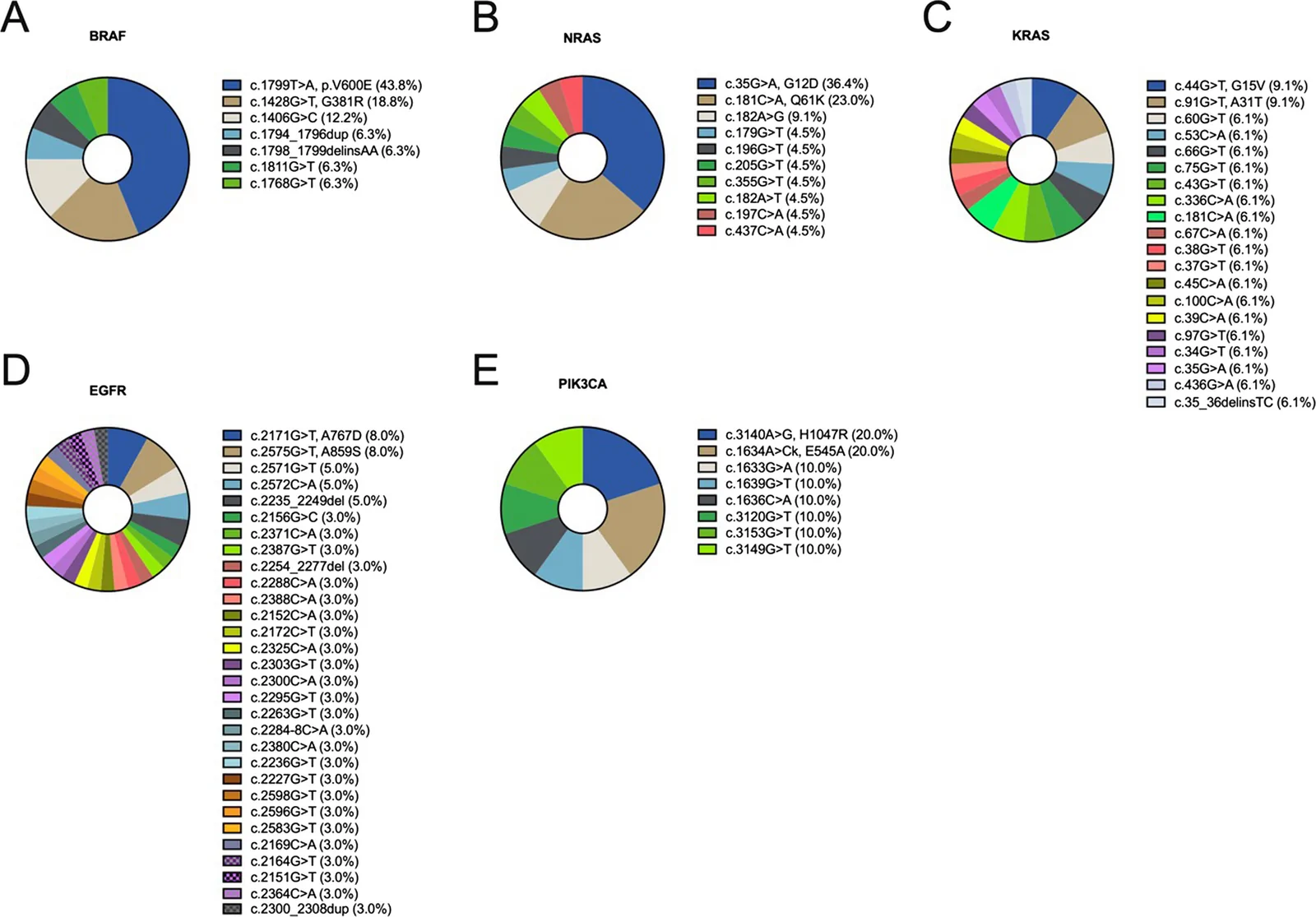
Summary of genetic variants and resulting amino acid changes in *BRAF* (**A**), *NRAS* (**B**), *KRAS* (**C**), *EGFR* (**D**), and *PIK3CA* (**E**) detected in ctDNA of all melanoma patients analyzed (*N* = 39)

cfDNA was extracted from all blood samples and analyzed by NGS; the results were then compared with 1,114 COSMIC-annotated mutations. In addition to the mutational status of the targeted five genes, we also obtained information about the coding DNA changes (CDS) and amino acid changes (AAC) per mutation. The detected CDS and the resulting protein changes in the ctDNA-positive patients are depicted in Fig. 1. The most frequently detected ctDNA mutations were *BRAF*^V600E^ (43.8%) and *NRAS*^G12D^ (36.4%), followed by *KRAS*^G15V^ and *KRAS*^A31T^ (both 9.1%), *EGFR*^A767D^ and *EGFR*^A859S^ (both 8.0%), and *PIK3CA*^E545A^ and *PIK3CA*^H1047R^ (both 20.0%).

### Analysis of ctDNA measurements at baseline

**Fig. 2:**
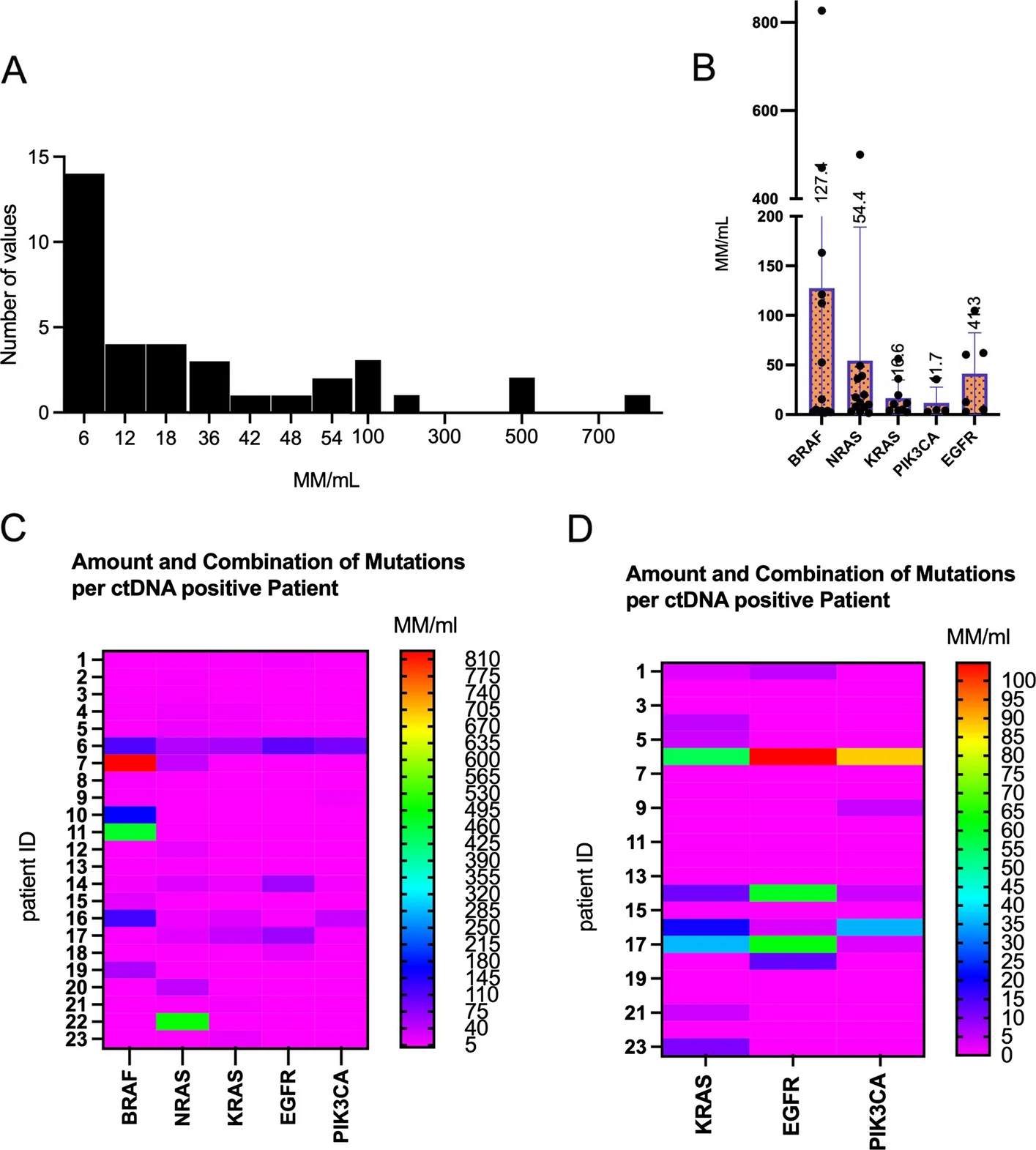
**A** Histogram showing the distribution of MM/mL amounts of all mutations; **B** Bar graph showing the amount of ctDNA in MM/mL per mutation (mean values) for *BRAF, NRAS, KRAS, PIK3CA,* and *EGFR*; **C** Amount and combination of all mutations detected in the five genes assessed per ctDNA positive patient; **D** Amount and combination of mutations in *KRAS, EGFR, PIK3CA* genes in ctDNA positive patients (zoom-in on novel-non-driver mutations)

Overall, 64.5% of patients at baseline were ctDNA positive (at least one of the five mutations detected). In peripheral blood, the median ctDNA level was 11.05 Mutant Molecules (MM) per mL (IQR 46.46 MM/mL), including the levels of all five mutations, with *BRAF* having the highest concentration with a mean of 127.4 MM/mL (standard deviation (SD) ± 237.7 MM/mL), followed by *NRAS* (54.4 MM/mL ± SD 134.8 MM/mL), *EGFR* (41.3 MM/mL ± SD 41.1 MM/mL), *KRAS* (16.6 MM/mL ± SD 18.3 MM/mL), and *PIK3CA* (11.7 MM/mL ± SD 15.96 MM/mL). The most frequent ctDNA concentrations varied between 5 and 40 MM/mL (Fig. 2A, 2B).

The heatmap shows the number and combination of mutations per patient at baseline. In total, 17.4% of patients harbored mutations in all five analyzed genes, 4.3% of patients had mutations in three out of five analyzed genes, 26.1% of patients had mutations in two of the studied genes, and 52.2% of patients had mutations in only one of the analyzed genes. *BRAF*-mutated ctDNA was detected in 42.4% of patients analyzed. In 15.2% of patients, *BRAF* was the only mutated gene, and in these patients, *the BRAF*^V600E variant^ was the only detected mutation. *NRAS* mutations were detected in 39.4% of patients and were the only mutation present in 12.1% of patients (*NRAS* ^G12D^). *KRAS* mutations were detected in 30.3% of patients and were the only mutations present in 6.1% of patients (*KRAS*^G15V^). *EGFR-mutated ctDNA* was detected in 18.2% of patients and occurs as a single mutation in 3.0% of patients (*EGFR*^A767D^). *PIK3CA* mutations were detected in 15.2% of patients and always co-occur in combination with *BRAF* or other mutations (Fig. 2C, 2D).

### Mutational landscape in tumor tissue and corresponding blood samples at baseline

**Fig. 3:**
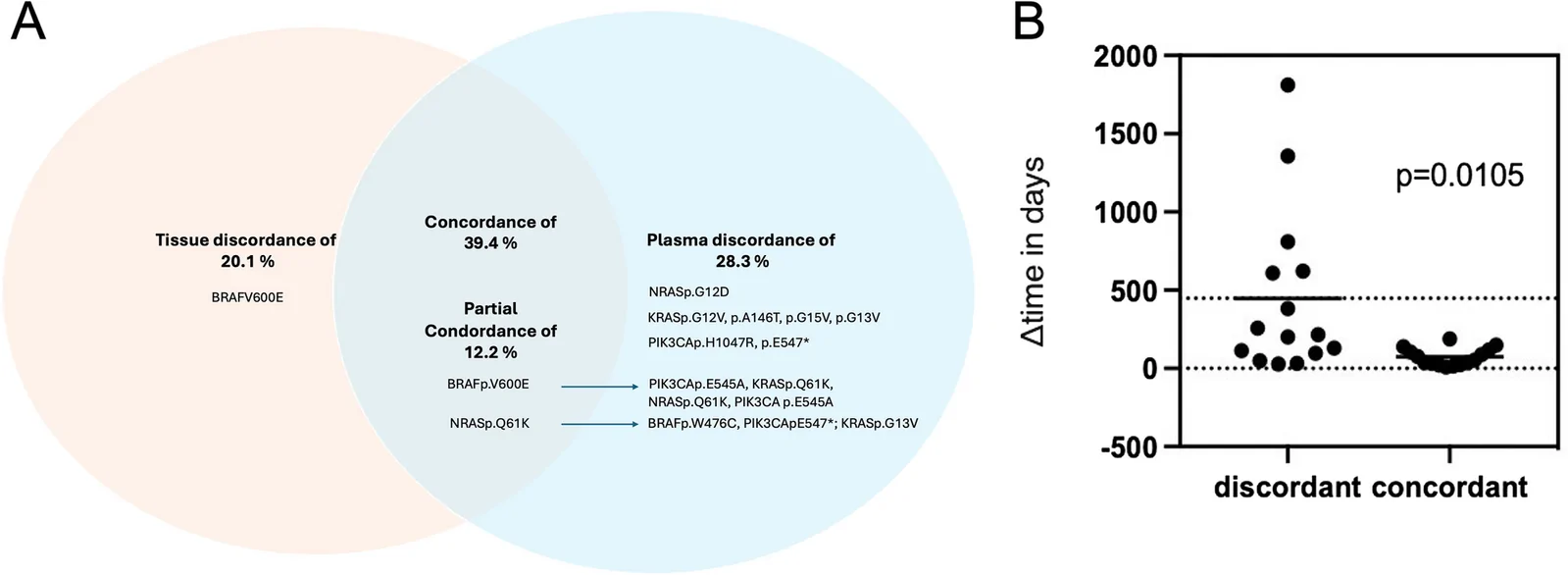
**A** Comparative analysis of mutations found in tumor tissue and plasma. Partial concordant findings include patients in whom matching and non-matching mutations were identified. Discordant findings include patients where either *BRAF* mutations were detected in tissue but not in blood or *BRAF, NRAS, KRAS* or *PIK3CA* mutations were detected in plasma but not in tissue; **B** An evaluation of the analysis time points and determination of the time difference between the tissue analysis and the liquid biopsy showed a significant correlation with concordance analyses and a short time delta of 73 days (*p* = 0.0105). “Concordant” findings include patients with complete and partial concordant findings

In the next step, we examined the concordance of the mutations detected in the tissue analysis with those detected by cfDNA analysis at the baseline (before the start of therapy). The overall concordance was 51.6%, including partial overlap where matching (*BRAF*^V600E^, *NRAS*^Q61K^) and non-matching mutations were found (Fig. 3A). For the discordant cases, we could distinguish three groups: One group had a *BRAF* mutation in the tissue that was not detectable in the blood at the start of therapy (20.1%). The second group had mutations (*BRAF, KRAS, NRAS, PIK3CA*) in the blood that were not detectable in the tissue (28.3%). The third patient group exhibited concordant mutations between tissue and blood but also harbored additional variants either within the concordant genes or in other genes (12.2%).

Tissue mutation analysis is often performed on the resected primary tumor. It may show an inevitable temporal delay compared to the liquid biopsy of mutations in blood taken before the start of therapy, depending on the time and the possibility of resection. We therefore analyzed the period between the resection date of the tumor tissue and the date of the liquid biopsy for each patient. We were able to show that in discordant patients, where the mutation status of the tissue does not match that of the blood, there is an average time difference of 448 days (SD ± 135.9 days). In comparison, patients with concordant findings, *i.e.* in which the mutation status of the tissue matches that of the blood, have an average time difference of 73 days (SD ± 56.03 days), which is significantly lower (p = 0.0105) compared to the mean time in discordant patients (Fig. 3B).

The detection of variants was not influenced by tumor content (which varied between 60 and 80% in all patient samples), and no significant correlation was observed between cfDNA concentration and ctDNA detection (*p* = 0.52) (Suppl. Figures 1, 2).

### Association of ctDNA measurements at baseline with risk factors and patients´ survival

We then examined the association between detectable ctDNA at baseline and melanoma-related clinical and pathological parameters (*N* = 31). There were no significant associations between ctDNA-positive and ctDNA-negative patients and their clinical and pathologic parameters (including sex, age, primary melanoma site, tissue mutations, AJCC, TNM). Moreover, no significant association with treatment parameters, including baseline treatment, number of treatment lines, and irAEs, was detected (Suppl. Table 1).

**Fig. 4:**
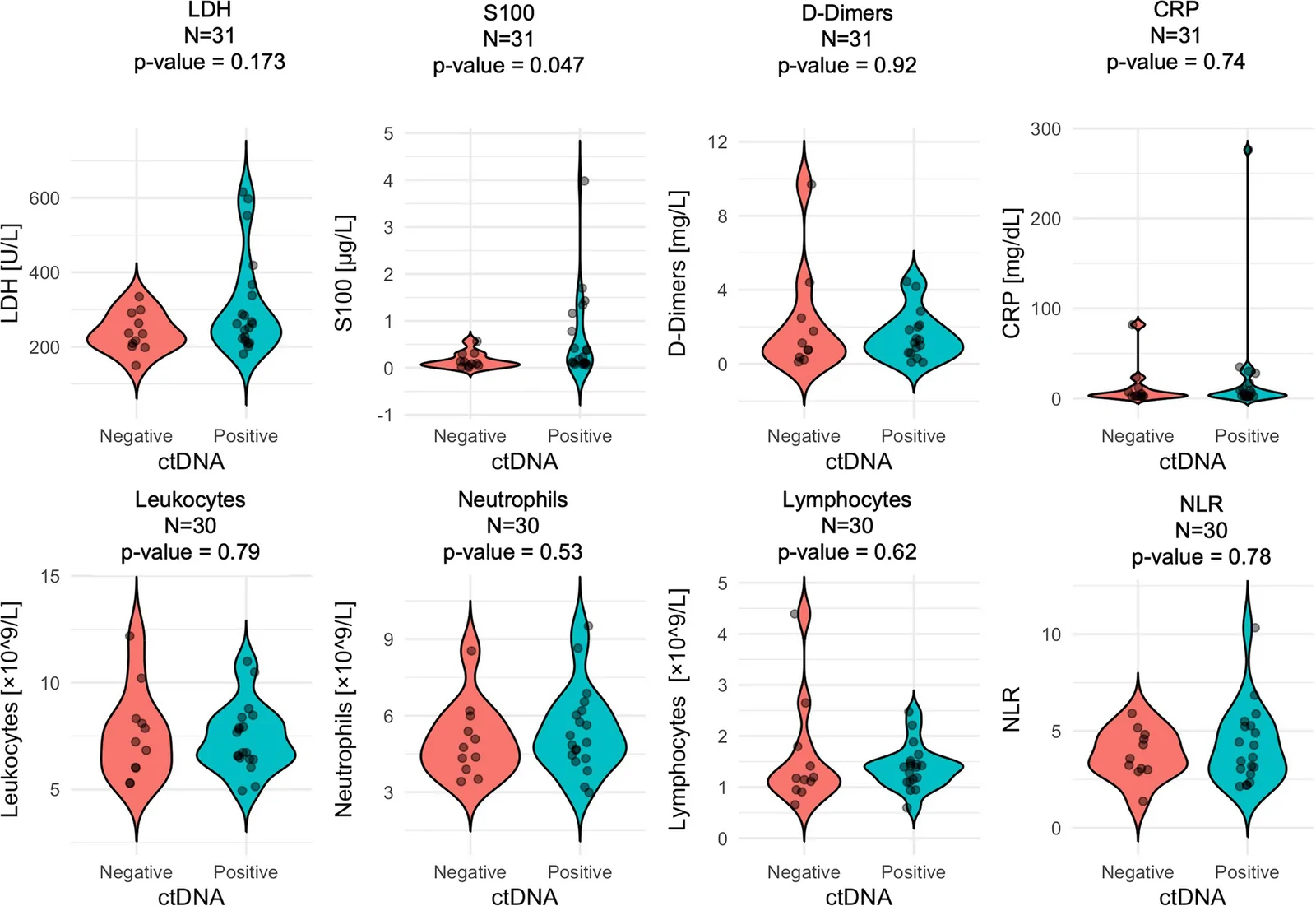
Violin plots (Mann–Whitney-U-test) of the baseline laboratory characteristics depending on the ctDNA findings; CRP, C-reactive protein; NLR, neutrophil/lymphocyte ratio

Additionally, we evaluated laboratory parameters that have previously been demonstrated to have prognostic value in melanoma. Serum concentrations of the tumor markers LDH and S100 were measured in all 31 patients. On average, ctDNA-positive patients showed a non-significant 1.5-fold higher LDH (332.5 U/L ± IQR 148.0 U/L vs. 259.8 U/L ± IQR 310.2 U/L; *p* = 0.173) and a significant 4.5-fold higher S100 (0.568 µg/L ± IQR 0.653 µg/L vs. 0.114 µg/L ± IQR 0.174 µg/L; *p* = 0.047) before treatment initiation. In addition, we examined leukocytes and their subsets relevant to melanoma progression (neutrophils, lymphocytes, and the neutrophil-to-lymphocyte ratio (NLR)), as well as the inflammation marker CRP. We did not detect any significant correlation with the other blood parameters (Fig. 4).

To assess the prognostic significance of baseline ctDNA measurements, Kaplan–Meier plots showing PFS and OS were created. Patients with detectable ctDNA showed a trend of borderline significance towards a shorter PFS (median PFS: 4 months [95% CI: 3–16] vs. 12 months [95% CI: 9- NA]) than patients without detectable ctDNA (*p* = 0.058) (Suppl. Figure 3). This difference was not yet seen for OS (Suppl. Figure 4).

### Longitudinal ctDNA analysis after initiation of therapy

**Fig. 5:**
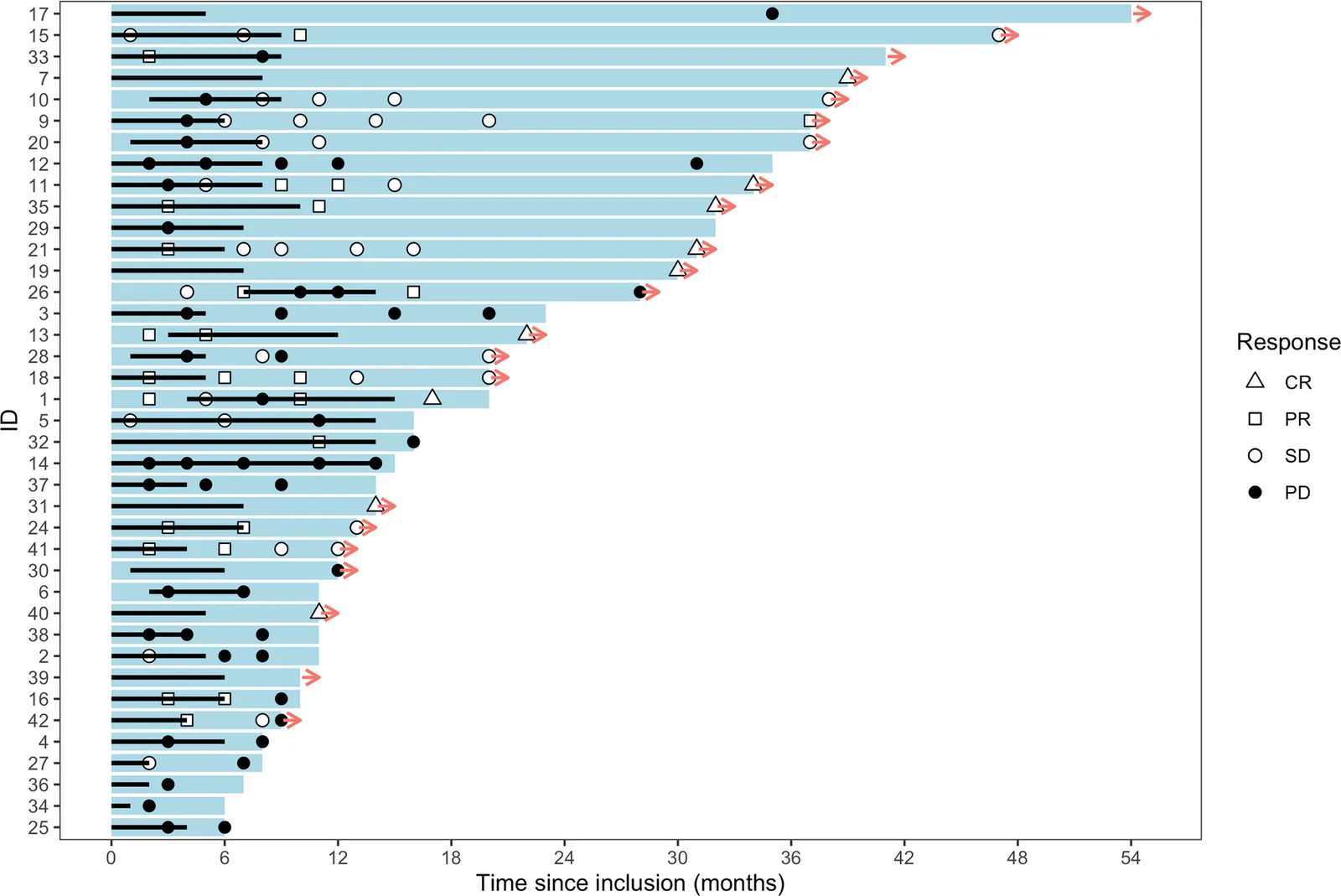
Swimmer plot showing the clinical course of all 39 patients included in the study. Each horizontal bar represents one patient and illustrates the duration of study inclusion. Arrows at the end of bars indicate patients who were still alive at the last follow-up. Black lines indicate the time frame in which ctDNA measurements were conducted. Radiological responses assessed by MRI (brain) and CT (thorax, abdomen, pelvis) every 3–4 months are indicated by different symbols: △ complete remission (CR), □ partial remission (PR), ○ stable disease (SD), and ● progressive disease (PD). The black bars represent longitudinal ctDNA measurements

Next, we analyzed the prognostic value of ctDNA measurements after initiation of treatment. To assess the dynamics of ctDNA under therapy, we analyzed serial measurements across multiple time points during the course of treatment (Fig. 5).

In total, 39 patients had available follow-up data and their associated treatment responses, as determined by routine cancer staging through medical imaging. Overall, 82.05% of patients were ctDNA positive at any time point (i.e., at least one mutation was detected in any of the five analyzed genes). We investigate the association and dynamics between these ctDNA levels and clinical parameters to evaluate molecular response patterns. This section presents key findings on ctDNA trends and their potential prognostic relevance under ICI treatment beyond baseline.

We analyzed the mutations identified at baseline and during follow-up to determine the best clinical response, as defined by RECIST criteria. The best overall response (BOR) was determined based on evaluation of all radiological staging assessments (up to the fifth staging), conducted approximately every 3–4 months. These clinical outcomes were then correlated with ctDNA levels to explore potential associations between molecular and radiographic response.

### ctDNA assessment after therapy initiation and association with best of response (BOR)

**Fig. 6:**
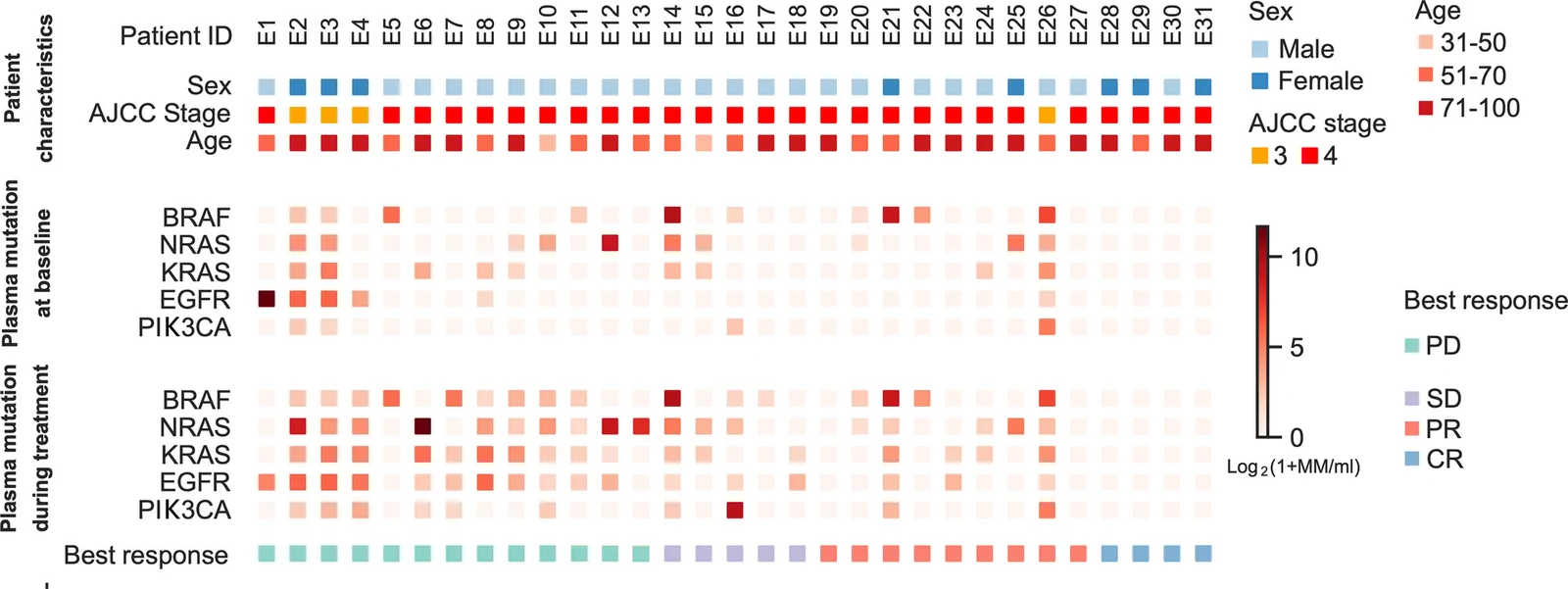
Heatmap showing patients’ main clinical and demographic characteristics and correlation of ctDNA in plasma to patients’ best response defined by radiological scans (cerebral MRI and CT of the thorax, abdomen, and pelvis)

The heat map in Fig. 6 shows the distribution of sex, stage, and age in the upper panel. The middle panel displays the mutations in the analyzed five genes at baseline and during therapy (*N* = 31). The lower panel shows the corresponding best radiological response, as determined by MRI of the brain and CT of the neck, thorax, and abdomen, including the pelvis. According to RECIST 1.1 Criteria, 41.9% of patients had a disease progression (PD), 16.1% of patients had a stable disease, and 42.0% of patients were responders (partial remission [PR, 29.1%] or complete remission [CR, 12.9%]). Of the 41.9% of PD-patients, 92.3% had mutations detected in their blood at baseline (#E1-#E6, #E8-#E13), 54.5% had *KRAS* mutations in combination or alone, followed by *NRAS* mutations (45.5%) and *BRAF* (36.4%). *PIK3CA* was detected in 18.3% of patients who developed a PD during treatment, in combination with a mutation in *BRAF* or all five genes.

In all patients with PD as BOR, ctDNA was detected during the course of treatment. When comparing the presence of specific mutations at baseline to their occurrence while on therapy, a notable increase was observed across all assessed genes. Taken together, the number of detected variants increased by 2.25-fold for *BRAF*, 2.0-fold for *NRAS*, 2.2-fold for *KRAS*, 2.4-fold for *EGFR,* and 2.0-fold for *PIK3CA* compared to treatment start. The ctDNA concentrations during treatment varied between 3–841 MM/mL (*BRAF*), 2–581 MM/mL (*NRAS*), 4–65 MM/mL (*KRAS*), 12–105 MM/mL (*EGFR*), and 2.6–8 MM/mL (*PIK3CA*).

In ten patients, no ctDNA was detected at baseline: one patient (#E7) shows PD congruent with detectable ctDNA during course of treatment, two patients (#E17, #E18) showed SD with detectable ctDNA during course of treatment, seven patients (#E19, #E23, #E27, #E28, #E29,#E30, #E31) showed response (PR or CR), and six of these seven patients remained ctDNA-negative under treatment.

We then analyzed ctDNA and serum biomarker levels collected during treatment of all 39 patients and stratified patients by their BOR into responders (PR/CR) and non-responders (PD). At baseline, ctDNA data (detectable and undetectable) were available for 79.5% (*N* = 31) of patients. Follow-up ctDNA values were available for 92.3% (*N* = 36) of patients at the timepoint of their first staging and for 79.5% (*N* = 31) at the timepoint of their second staging.

**Fig. 7:**
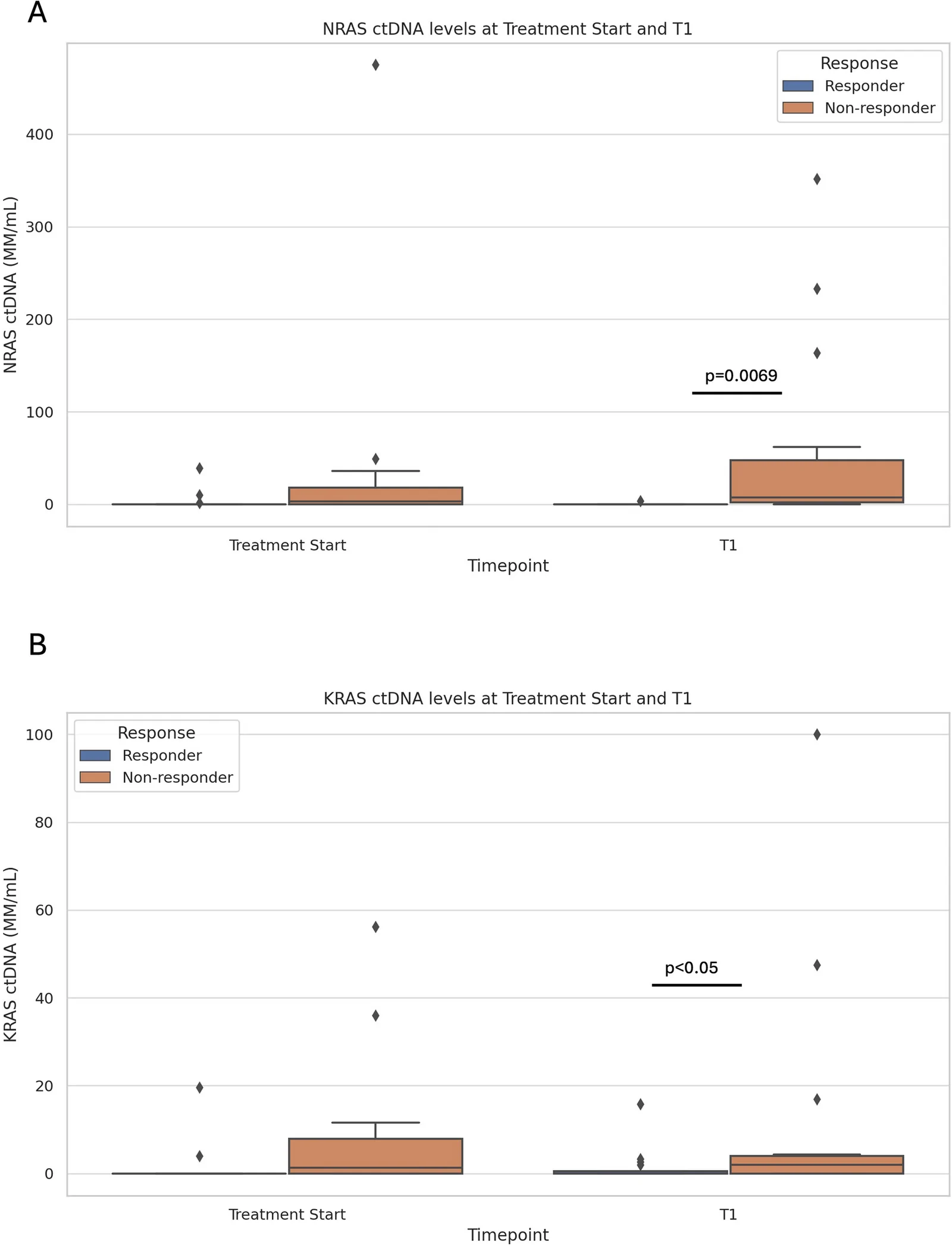
Mean *KRAS/NRAS* ctDNA levels (MM/mL) at Start of treatment and T1 in responders (PR/CR) and non-responders (PD). Due to non-normal distribution and presence of outliers in ctDNA levels, median values and interquartile ranges were used to summarize central tendency. Boxplots show median and interquartile range (IQR); outliers are displayed as individual points.; **A** *NRAS* ctDNA levels at T1 were significantly higher in non-reponders compared to responders (*p* = 0.0069, Mann–Whitney U test); **B** *KRAS* ctDNA levels at T1 were significantly higher in non-responders compared to responders (*p* = 0.028, Mann–Whitney U test)

At baseline, none of the eight biomarkers (*BRAF*^*mutated*^ ctDNA, *NRAS*^*mutated*^ ctDNA; *KRAS*^*mutated*^ ctDNA, *EGFR*^*mutated*^ ctDNA, *PIK3CA*^*mutated*^ ctDNA, S100, LDH, D-dimers) and BOR were significantly different between response groups (Suppl. Figure 5). In contrast, for blood samples analyzed at the first clinical response assessment (T1), the detection of several ctDNA mutations showed a stronger association with clinical outcome. *NRAS* mutation levels in ctDNA were significantly higher in non-responders with progressive disease (PD) compared to responders (PR/CR) (median 15.9 MM/mL vs. 0 MM/mL, IQR 40.5 MM/mL, *p* = 0.0069). Similarly, *KRAS* mutation levels were elevated in non-responders compared to responders (median 7.5 MM/mL vs. mean 0 MM/mL, IQR 45.3 MM/mL, *p* = 0.028) (Fig. 7A,7B).

### ctDNA assessment after therapy initiation and survival

**Fig. 8:**
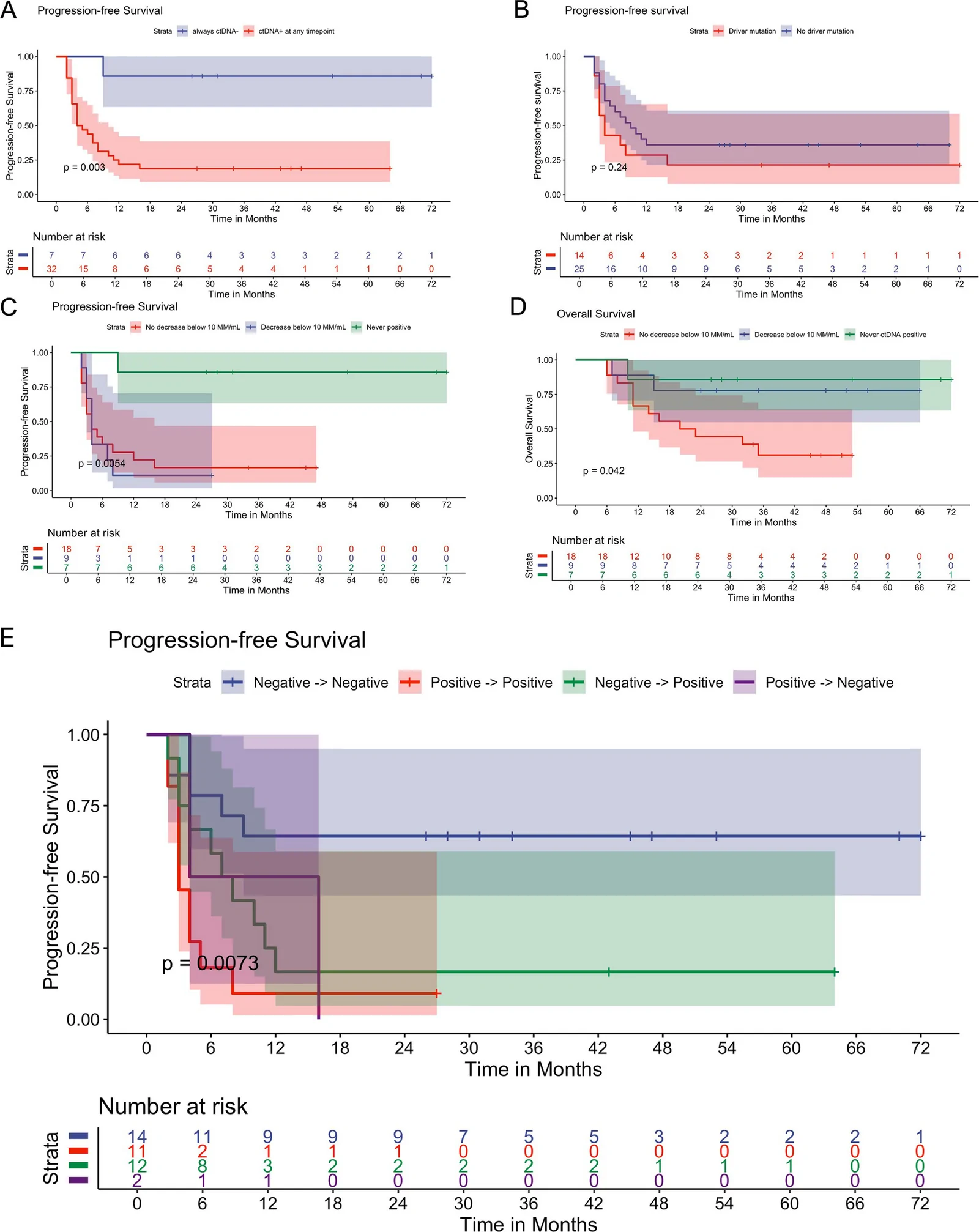
**A** Kaplan–Meier plot showing the progression-free survival (PFS) probability of ctDNA-positive (at least one mutation detected) and ctDNA-negative patients (*p* = 0.003); **B** Kaplan–Meier plot showing the progression-free survival (PFS) between patients with driver (red) vs. non-driver (blue) mutations (*p* = 0.24). **C** Kaplan–Meier plot showing the progression-free survival (PFS) between (green) patients that were never ctDNA positive, (blue) patients with a decrease lower than 10 MM/mL, and (red) patients without a decrease below 10 MM/mL during treatment (*p* = 0.0054). **D** Kaplan–Meier plot showing the overall survival (OS) between (green) patients that were never ctDNA positive, (blue) patients with a decrease below 10 MM/mL, and (red) patients without a decrease below 10 MM/mL during treatment (*p* = 0.042). **E** Kaplan–Meier plot showing the progression-free survival (PFS) probability of four patients’ groups: (blue) ctDNA negative stay negative, (green) ctDNA negative become positive, (red) ctDNA-positive stay positive, and (purple) ctDNA positive that became negative (*p* = 0.0073)

To assess the prognostic significance of ctDNA during treatment, Kaplan–Meier plots showing PFS and OS were created. Patients who were ctDNA-positive at any time point during the study exhibited a significantly shorter median PFS (median 4.5 months [95% CI: 4–10]) compared to patients who remained ctDNA-negative (median not reached) throughout follow-up (p = 0.003) (Fig. 8A). At the same time, there was no significant difference in OS between the groups (*p* = 0.15, Suppl. Figure 6).

Given that the NGS-based assay used in this study enables the detection of hundreds of mutations per gene, we investigated the prognostic value for PFS and OS of less frequent mutations compared to the most frequent mutations within the patient cohort. Thus, the ctDNA-positive patients were divided into two groups: driver mutations (defined as the two most frequent variants at all measured time points in this study) and non-driver mutations (defined as the least frequent variants at all measured time points). For both PFS (Fig. 8B) and OS (Suppl. Figure 7), the analysis of driver versus non-driver mutations showed no significant difference (*p* = 0.24 and *p* = 0.97, respectively), indicating unfavorable clinical outcomes regardless of whether any mutation is present.

Next, we subsequently investigated whether very low amounts of ctDNA are relevant for predicting clinical outcome. Patients were stratified into three groups based on ctDNA measurements during treatment: never ctDNA-positive, ctDNA-positive with a decrease to less than the absolute value of 10 MM/mL, and ctDNA-positive > 10 MM/mL. The latter group includes patients who have ctDNA levels greater than 10 MM/mL, who have never reached a ctDNA concentration of less than the absolute value of 10 MM/mL during measurement. Kaplan–Meier analysis revealed a statistically significant difference in PFS between these groups (*p* = 0.0054). Patients who were never ctDNA-positive demonstrated the most favorable outcomes (median not reached), with long-term PFS. In contrast, both groups with detectable ctDNA, regardless of the extent of change in ctDNA concentration, showed markedly worse PFS (< = 10 median 4 months [95% CI: 3-NA] and > 10 MM/mL (median 4 months [95% CI: 3–16]) (Fig. 8C). Concerning OS, patients with higher ctDNA levels than 10 MM/mL appeared to have poorer survival (median 21.5 month [95% CI: 11-NA]) than consistently ctDNA negative patients (median not reached) and patients with ctDNA levels below 10 MM/mL (median not reached) (*p* = 0.042) **(**Fig. 8D**).**

Given the potential of ctDNA dynamics to reflect early treatment response, we examined the association between dynamic ctDNA trajectories after therapy initiation and clinical outcomes, including PFS and OS. Rather than focusing on a single time point, we aimed to capture the temporal patterns of ctDNA changes under treatment. PFS significantly differed according to ctDNA measurements during treatment (Fig. 8E). Kaplan–Meier analysis showed that patients who remained ctDNA-negative throughout the course had the most favorable PFS (median not reached)in comparison to patients with persistent ctDNA positivity (median 3 months [95% CI: 3-NA]), or those who converted from negative to positive (median 7.5 months [95% CI: 4-NA]), exhibited significantly shorter PFS (*p* = 0.0073). Surprisingly, the two patients with a ctDNA-positive to ctDNA-negative change also had an impaired PFS (median, 10 months [95% CI: 4- NA]). Inspection of the patient records showed that they had cerebral metastases that may be responsible for the impaired PFS independent of the status as ctDNA negative (Fig. 8E).

### Association of Genetic ctDNA Variants with Metastatic Sites

**Fig. 9:**
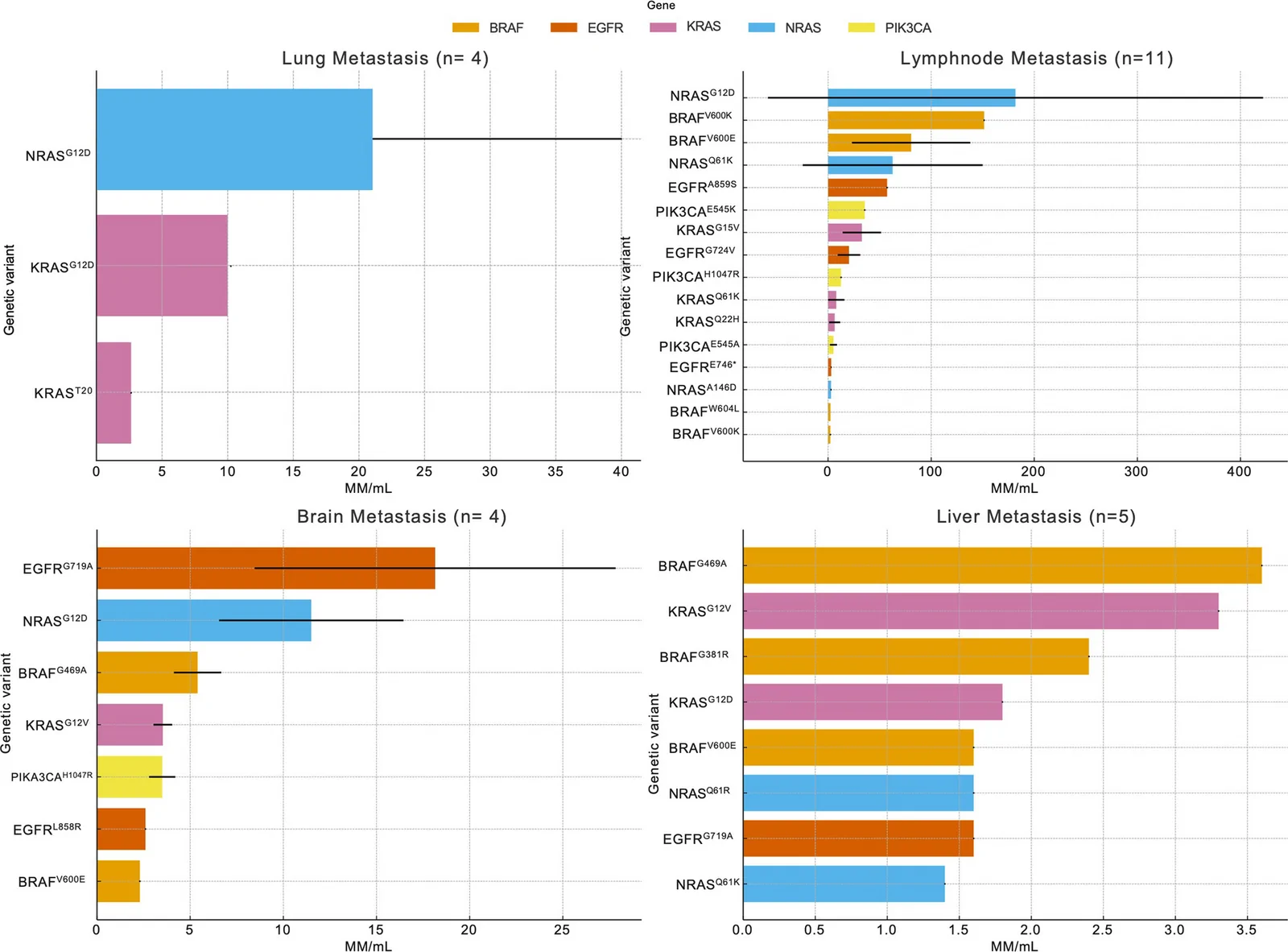
Mean mutation burden (MM/mL) of individual genetic variants stratified by metastatic site. Bars indicate the average mutation level per variant in each organ site (lung, lymph nodes, brain, liver), color-coded by gene (e.g., *BRAF*, *EGFR*, *KRAS*, *NRAS*, *PIK3CA*). Variants such as KRAS^*G12D*^, EGFR^*G719A*^, and BRAF^*G469A*^ show site-specific patterns, suggesting differential representation across metastatic environments

The mutation load of individual genetic ctDNA variants was compared across different single metastatic sites at baseline and during the course of treatment. In patients with lung metastases, *KRAS*^G12D^ and NRAS^G12D^ ctDNA mutations were the most frequently detected. Additionally, *EGFR*^G719A^ and *NRAS*^G12D^, as well as *BRAF*^G469A^, were also enriched in patients with brain metastases. Furthermore, KRAS^G12V^ was enriched in patients with brain metastases. In patients with liver metastases, *BRAF*^G469A^, *KRAS*^G12V^, and *KRAS*^G12D^ were most frequently detected in blood. Patients with only lymph node metastases showed a broader spectrum, with contributions from multiple pathogenic variants of *EGFR*, *BRAF*, and *NRAS* **(**Fig. 9**).**

**Fig. 10:**
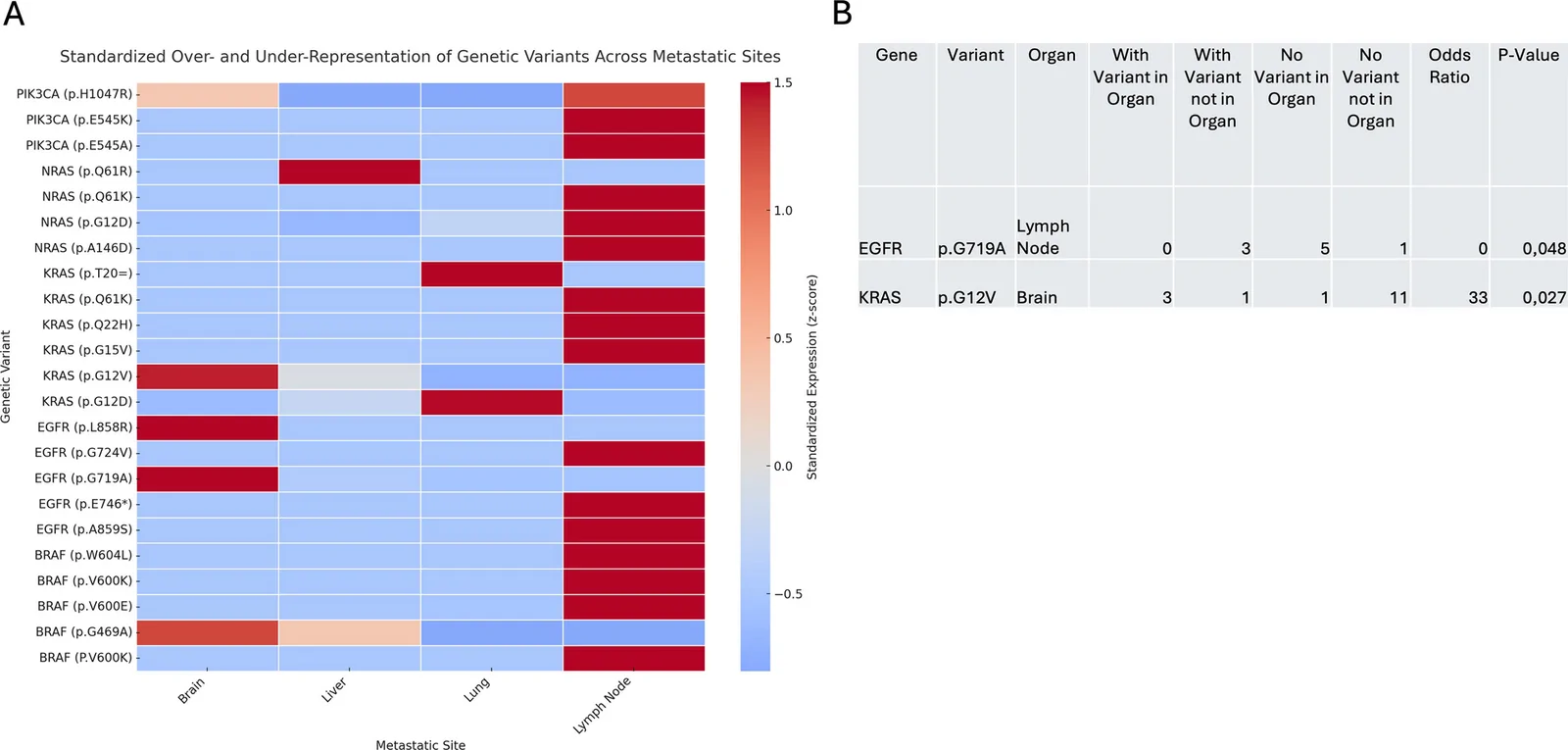
**(A)** Heatmap showing standardized over- and under-representation of genetic variant concentrations in ctDNA across metastatic sites. Each row represents a specific genetic variant, and each column represents a metastatic site (lung, liver, lymph node, brain). Colors indicate standardized expression levels (z-scores): red for over-representation, blue for under-representation, and white for average levels. **(B)** Results from Fisher’s Exact Test indicate statistically significant associations (*p* < 0.05) between specific genetic variants and metastatic organ sites. The mutation *KRAS*^G12V^ is overrepresented in brain metastases, with a significantly higher odds ratio (33.00, *p* = 0.027)

The heatmap illustrates patterns such as *BRAF*^G469A^ being more prevalent in liver metastases and *PIK3CA*^H1047R^ in brain metastases, while *KRAS*^G12D^ was predominantly observed in lung metastases (Fig. 10A). Additionally, specific genetic alterations were found to have a clear association with particular locations. For example, *KRAS*^G12V^ was notably more common in brain metastases (*p* = 0.027), whereas *EGFR* ^G719A^ appeared less frequently in lymph node metastases (*p* = 0.048) (Fig. 10B). Additionally,

## Discussion

This study demonstrates the utility of a targeted NGS-based approach for detecting ctDNA mutations in advanced melanoma patients undergoing immune checkpoint inhibition (ICI). By focusing on hotspot regions in five genes (*BRAF, EGFR, KRAS*, *NRAS*, and *PIK3CA*), we showed that ctDNA is detectable in a high proportion of patients and provides valuable prognostic information. These findings underscore the molecular complexity of melanoma and emphasize the need for highly sensitive and repetitive detection strategies to capture tumor dynamics in real-time.

A substantial proportion of patients were ctDNA-positive at baseline (65.5%) and across all time points (82.05%), supporting ctDNA as a non-invasive biomarker for real-time tumor monitoring. Mutations in all five genes were identified, underscoring the genetic heterogeneity of melanoma. *BRAF* mutations, especially *BRAF*^V600E^, were most prevalent, consistent with previous literature [12]. However, 39.1% of samples lacked *BRAF* mutations, emphasizing the necessity for broader genomic panels to enhance detection.

*KRAS* and *NRAS* mutations were detected in 30.3% and 39.4% of patients, respectively, and were more frequent among non-responders, suggesting a potential role in resistance to treatment. Variants such as *KRAS* ^G13D^ and *KRAS* ^G12D^ were found in 54.5% of non-responders, consistent with aggressive disease phenotypes [13, 14]. While *KRAS* mutations are less frequent in melanoma compared to colorectal or pancreatic cancers, their impact on progression and therapy resistance is increasingly recognized [15].

*EGFR* signaling has been implicated in melanoma progression through alternative mechanisms such as copy number variations and splice variants [16, 17]. These alterations appear to be particularly relevant in acral and mucosal melanomas, which are underrepresented in the TCGA’s cutaneous melanoma cohort, but show a higher prevalence of *EGFR* and *PIK3CA* mutations [18]. In our cohort, rare *EGFR* mutations, such as *EGFR* ^859S^ and *EGFR*^767D^, were detected in 16% of *EGFR*-positive cases, often in combination with other genomic alterations. Although these mutations are well-established in non-small cell lung cancer (NSCLC) and colorectal cancer [19, 20], their role in melanoma remains poorly defined. The observed association with progressive disease (PD) and stable disease (SD) in our cohort suggests the existence of *EGFR*-driven melanoma subtypes, warranting further investigation into the functional consequences of *EGFR* activation [17, 21].

In addition, *PIK3CA* mutations, typically rare in melanoma (< 5%) [22], were observed in 21.7% of our patients. Most were gain-of-function mutations (e.g., E545K, H1047R), which are known to confer resistance to MEK and CDK4/6 inhibitors in *NRAS*-mutant melanoma [23–25]. Their association with PD in our cohort highlights *PIK3CA* as a potential therapeutic target, especially given the availability of selective *PIK3CA* inhibitors in other malignancies [25].

Baseline ctDNA was significantly associated with elevated S100 levels but not with age, gender, or treatment (Suppl. Table 1). Combining ctDNA and S100 may improve prognostic accuracy. Notably, ctDNA positivity after therapy initiation correlated with significantly shorter PFS (Fig. 8A), while baseline ctDNA levels alone showed only a trend toward worse survival (Suppl. Figures 3, 4). This supports the added value of longitudinal, dynamic monitoring over single-time-point measurements.

At baseline, *NRAS* and *KRAS* mutation levels were not significantly different between responders and non-responders. However, at the first clinical assessment, the ctDNA levels of these mutations were significantly higher in non-responders. This temporal difference underscores the importance of early on-treatment monitoring to predict clinical response and further highlights the need for repeated, sensitive ctDNA measurements. These findings align with prior reports showing that ctDNA provides a more accurate and dynamic reflection of tumor burden than serum markers such as S100 alone [26–29].

Patients who remained ctDNA-positive or converted to positive status during treatment exhibited worse PFS. Interestingly, two patients who converted to ctDNA-negative still progressed rapidly due to cerebral metastases. These cases likely reflect the limited shedding of ctDNA from brain metastases and primary central nervous system tumors [30, 31], underscoring a key limitation of plasma-based ctDNA analysis.

We observed a moderate concordance rate (51.6%) between tissue and plasma mutation profiles, which contrasts with prior reports of greater than 90% concordance in prospective cohorts [32]. This discrepancy likely reflects the time difference between tissue biopsy and plasma sampling. Patients with discordant findings had a mean difference of 448 days between sampling compared to 73 days in concordant cases. These findings suggest that ctDNA analysis provides a more up-to-date snapshot of tumor biology and is particularly useful under ongoing therapeutic pressure, consistent with observations in non-small cell lung cancer (NSCLC) [33]. Notably, 12.2% of patients showed partial concordance, in which the same mutations were detected in both tissue and plasma, but with additional mutations in different genes detected exclusively in plasma. This suggests that blood-based analysis not only complements tissue-based diagnostics but may also reveal emerging or subclonal alterations that are not captured in archived tumor biopsies.

Both hotspot and non-hotspot mutations were associated with poor PFS, suggesting that ctDNA reflects overall tumor activity rather than the presence of canonical driver mutations alone. Panels limited to hotspot regions may, therefore, miss relevant alterations, leading to misclassification of patients.

Furthermore, even modest reductions in ctDNA levels (< 10 MM/mL) did not translate into improved prognosis. This emphasizes the prognostic value of minimal residual ctDNA and supports the development of highly sensitive assays capable of detecting low-frequency mutations.

Distinct mutational patterns were observed across metastatic sites. *KRAS*^G12D^ was enriched in lung metastases, *BRAF*^G469A^ and *NRAS*^Q61R^ were more common in liver metastases, and* KRAS*^G12V^ appeared predominantly in brain lesions. These site-specific associations may reflect microenvironmental selection pressures or differing immune surveillance, but they remain hypothesis-generating and require functional validation [34–36]. These findings further emphasize the molecular complexity of melanoma and suggest that understanding the interaction between mutational profile and metastatic behavior could inform tailored therapeutic approaches in the future.

Limitations of this study include the small sample size (*N* = 39) and the single-center, real-world setting, which may limit the generalizability of the findings. The homogeneity in staging and treatment enhances internal consistency, but subgroup analyses should be interpreted cautiously. Additionally, the limited gene panel did not include emerging melanoma drivers, such as *TERT* or *NF1* [37–39], which could improve detection and stratification in future studies.

This study highlights the prognostic value of ctDNA monitoring in advanced melanoma, demonstrating that dynamic assessments offer superior insight compared to baseline measurements. Our data support the role of ctDNA in capturing ongoing tumor evolution, suggesting that specific mutations, including rare variants in *KRAS, EGFR,* and *PIK3CA,* may contribute to resistance and progression.

Targeted ctDNA panels—although less personalized than tumor-informed approaches—offer practical advantages, particularly in clinical settings where access to tumor tissue is limited. Future work should compare tumor-informed and tumor-agnostic assays in prospective, site-specific trials and evaluate whether ctDNA-guided therapeutic switching improves patient outcomes.

Overall, our findings underscore the need for comprehensive and sensitive ctDNA profiling strategies to capture the molecular complexity of melanoma and facilitate more personalized treatment approaches. These insights support the integration of ctDNA monitoring into clinical workflows and warrant further exploration in larger, interventional trials.

## Supplementary Information

Supplementary Material 1. Data

## Data Availability

The majority of the data generated or analyzed during this study are included in this published article and its supplementary information files. Part of the data generated in this study is not publicly available due to patient privacy requirements, but is available upon reasonable request to the corresponding author.

## Abbreviations

ctDNA: Circulating tumor DNA
ICI: Immune Checkpoint Inhibition
LB: Liquid biopsy
WGS: Whole Genome Sequencing
WES: Whole Exome Sequencing
PCR: Polymerase chain reaction
ddPCR: Digital droplet PCR
NGS: Next generation sequencing
AJCC: American Joint Committee on Cancer
T: Tumor
N: Nodes
M: Metastases
IQR: Interquartile range
SD: Standard deviation
MM: Mutant molecules
CDS: Coding DNA Sequence
AAC: Amino Acid Changes
LDH: Lactate dehydrogenase
S100: S100 protein
RECIST: Response Evaluation Criteria in Solid Tumors
MRI: Magnetic resonance imaging
CT: Computer tomography
PD: Progressive disease
MR: Mixed response
SD: Stable disease
PR: Partial remission
CR: Complete remission
NED: No evidence of disease
PFS: Progression free survival
OS: Overall survival
MAF: Mutant allele fraction
NSCLC: Non-small cell lung cancer
Human Genes: BRAF: B-Raf proto-oncogene
NRAS: NRAS proto-oncogene
KRAS: Kirsten rat sarcoma virus
EGFR: Epidermal growth factor receptor
PIK3CA: Phosphatidylinositol-4,5-bisphosphate 3-kinase catalytic subunit alpha
