# Supplementary material for: In-depth assessment of BRAF, NRAS, KRAS, EGFR, and PIK3CA mutations on cell-free DNA in the blood of melanoma patients receiving immune checkpoint inhibition
Source: J Exp Clin Cancer Res. 2025 Jul 12;44:202. doi: 10.1186/s13046-025-03457-w (PMC12255093; doi:10.1186/s13046-025-03457-w)
Supplement: Supplementary file 1 — Supplementary Material 1. Data [file 13046_2025_3457_MOESM1_ESM.docx]

**Supplemental Data**

Table 1: Association of ctDNA measurements at baseline to clinical, biochemical, and pathological risk factors

| ctDNA at baseline | Total N |  | Negative | Positive | p |
| --- | --- | --- | --- | --- | --- |
| Sex | 31 (100.0) | Male | 8 (72.7) | 15 (75.0) | 1.000 |
|  |  | Female | 3 (27.3) | 5 (25.0) |  |
| Age group | 31 (100.0) | <65 | 2 (18.2) | 8 (40.0) | 0.262 |
|  |  | ≥65 | 9 (81.8) | 12 (60.0) |  |
| Primary melanoma site | 31 (100.0) | Cutaneous | 9 (81.8) | 19 (95.0) | 0.281 |
|  |  | Mucosal | 2 (18.2) | 1 (5.0) |  |
| BRAF status (tissue) | 30* (96.8) | Wildtype | 6 (54.5) | 11 (57.9) | 0.644 |
|  |  | BRAF V600E | 4 (36.4) | 8 (42.1) |  |
|  |  | BRAF V600K | 0 (0.0) | 0 (0.0) |  |
|  |  | BRAF V600R | 1 (9.1) | 0 (0.0) |  |
| KRAS status (tissue) | 29* (93.5) | Wildtype | 11 (100.0) | 17 (94.4) | 1.000 |
|  |  | Mutated | 0 (0.0) | 1 (5.6) |  |
| NRAS status (tissue) | 30* (96.8) | Wildtype | 10 (90.9) | 16 (84.2) | 1.000 |
|  |  | NRAS Q61R | 1 (9.1) | 3 (15.8) |  |
|  |  | NRAS Q61K | 0 (0.0) | 0 (0.0) |  |
| AJCC | 31 (100.0) | Stage III | 0 (0.0) | 4 (20.0) | 0.269 |
|  |  | Stage IV | 11 (100.0) | 16 (80.0) |  |
| T | 31 (100.0) | T0 | 4 (36.4) | 4 (20.0) | 0.350 |
|  |  | T1 | 0 (0.0) | 3 (15.0) |  |
|  |  | T2 | 0 (0.0) | 3 (15.0) |  |
|  |  | T3 | 1 (9.1) | 2 (10.0) |  |
|  |  | T4 | 6 (54.5) | 6 (30.0) |  |
|  |  | Tx | 0 (0.0) | 2 (10.0) |  |
| N | 31 (100.0) | N0 | 3 (27.3) | 6 (30.0) | 0.336 |
|  |  | N1 | 1 (9.1) | 7 (35.0) |  |
|  |  | N2 | 3 (27.3) | 4 (20.0) |  |
|  |  | N3 | 4 (36.4) | 3 (15.0) |  |
| M | 31 (100.0) | M0 | 1 (9.1) | 4 (20.0) | 0.631 |
|  |  | M1 | 10 (90.9) | 16 (80.0) |  |
| Baseline therapy | 31 (100.0) | Ipilimumab + Nivolumab | 7 (63.6) | 12 (60.0) | 1.000 |
|  |  | Pembrolizumab | 3 (27.3) | 6 (30.0) |  |
|  |  | Nivolumab | 1 (9.1) | 2 (10.0) |  |
| No. of treatment lines | 31 (100.0) | First line | 8 (72.7) | 16 (80.0) | 0.768 |
|  |  | Second line | 3 (27.3) | 3 (15.0) |  |
|  |  | Third line | 0 (0.0) | 1 (5.0) |  |
| Immune-related adverse events | 29** (93.5) | No | 8 (72.7) | 8 (44.4) | 0.249 |
|  |  | Yes | 3 (27.3) | 10 (55.6) |  |

* The patients missing here had not enough tissue for mutational analysis.
** The missing information on the irAE status was not available.

EGFR analysis in tissue was not performed. T Tumor; N, lymph nodes; M, distant metastasis; according to the 8th edition of the AJCC cancer staging manual.

**Suppl. Figure 1:** Histogram of tumor content across all patient samples. Tumor content ranged from 60% to 80%, with the majority of samples showing values between 65% and 75%. The detection of genetic variants was not influenced by tumor content within this range.

**Suppl. Figure 2:** Comparison of total circulating DNA (pg/DNA) between ctDNA-positive and ctDNA-negative samples.Boxplot showing pg/DNA levels by ctDNA status. Mean values are marked with white circles; individual values are shown as black dots. No statistically significant difference was observed between the groups (Welch’s t-test, *p* = 0.52).


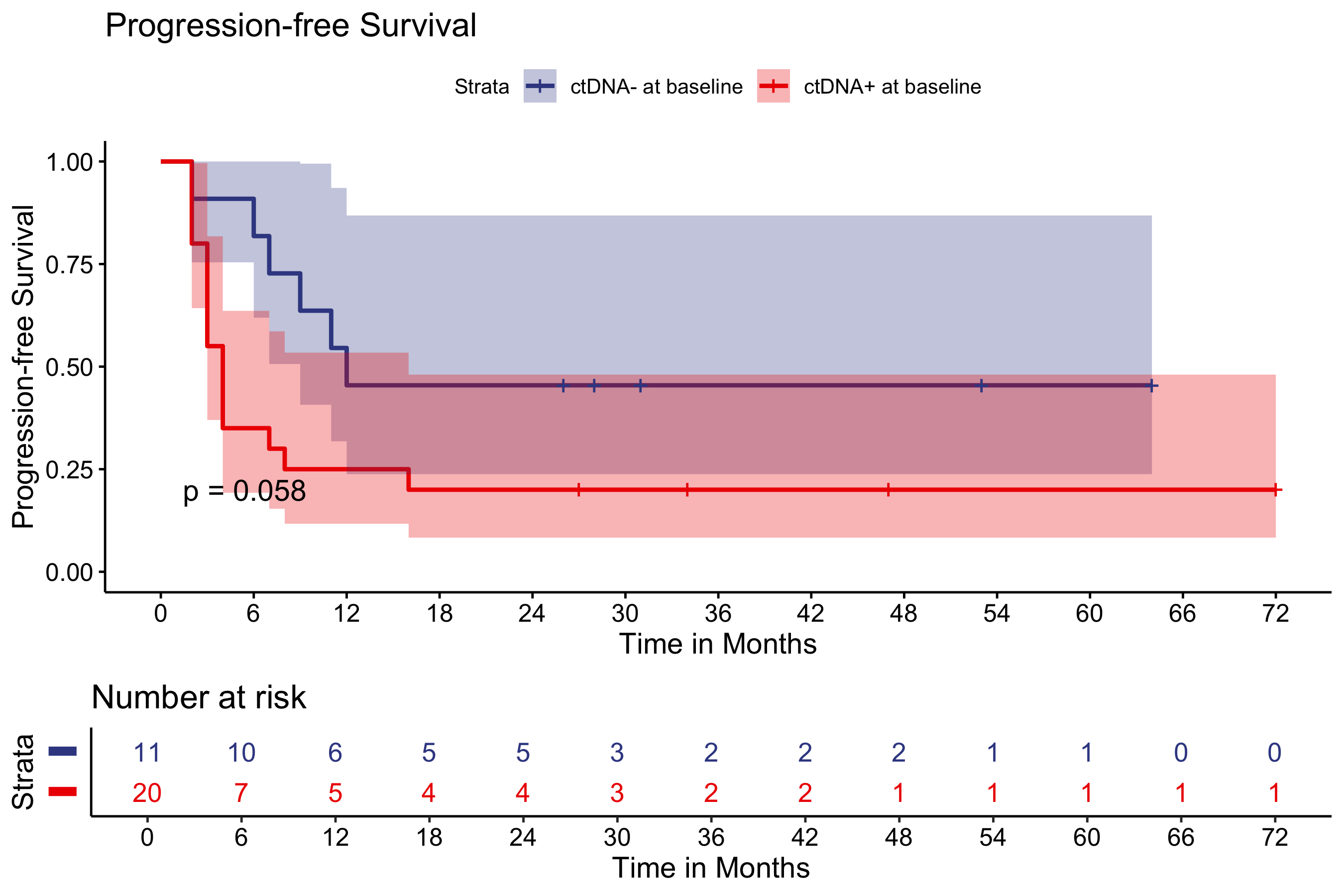


**Suppl. Figure 3:** Kaplan–Meier plot showing the progression-free survival (PFS) probability of ctDNA-positive (at least one mutation detected) and ctDNA-negative patients before treatment (at baseline) (p=0.058) (N=31)


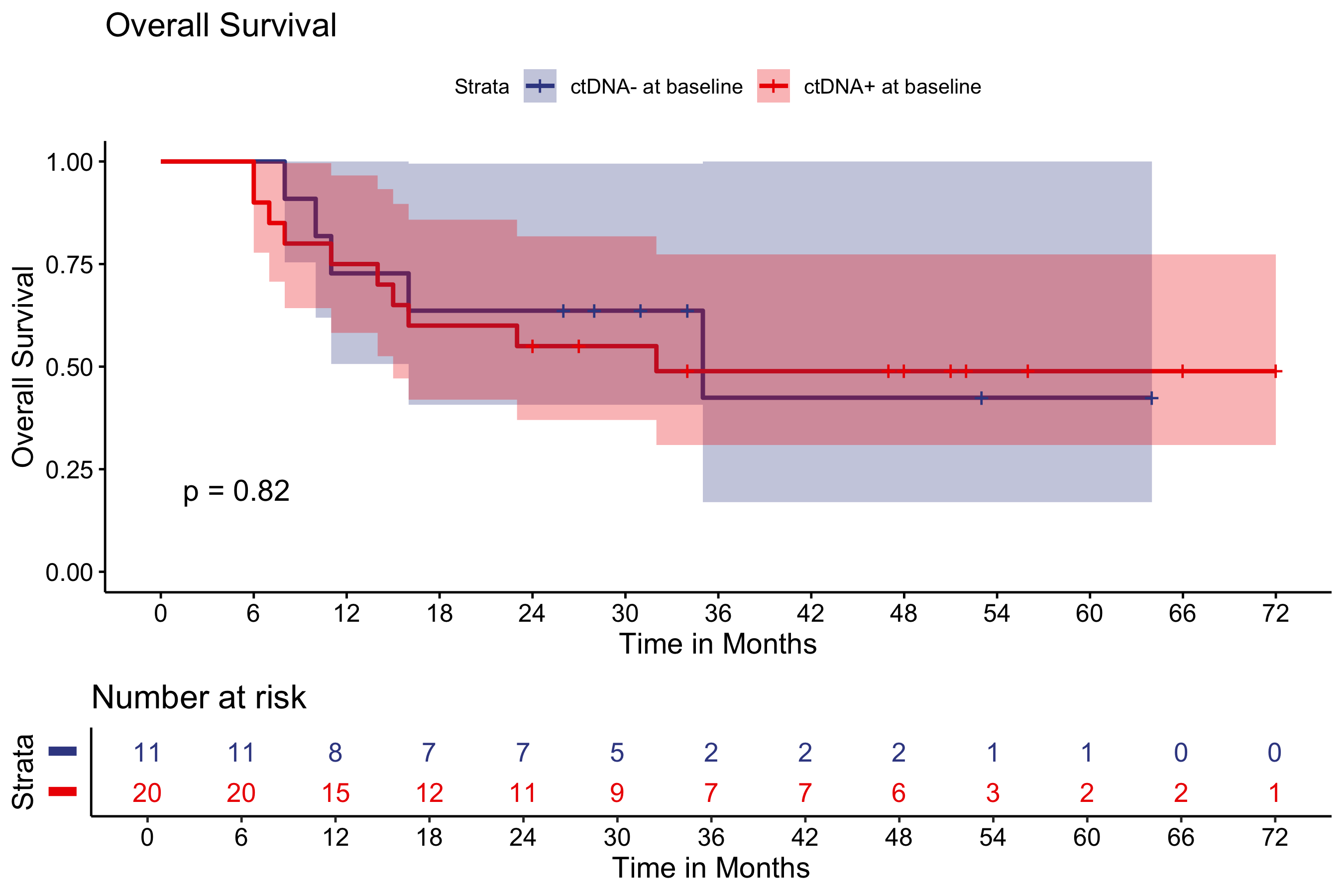


**Suppl. Figure 4:** Kaplan–Meier plot showing the overall survival (OS) probability of ctDNA-positive (at least one mutation detected) and ctDNA-negative patients before treatment (at baseline) (p=0.82)(N=31)


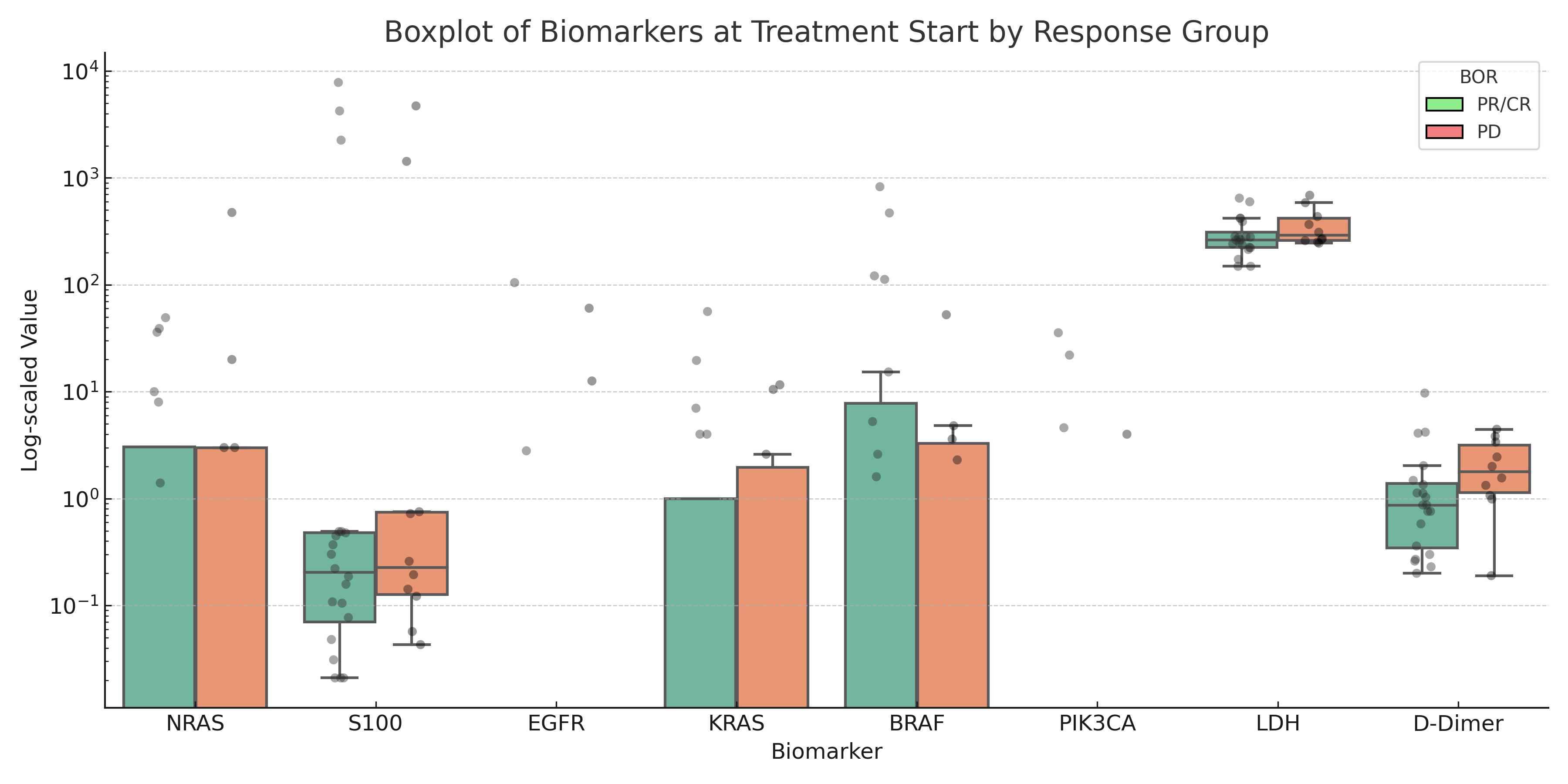


**Suppl. Figure 5:** Boxplot showing the distribution of ctDNA in MM/mL (*BRAF, NRAS, KRAS, EGFR, PIK3CA*) and serum biomarkers (LDH U/L, S100 µg/L, D-Dimer mg/L) at the start of treatment, grouped by response (PR/CR vs. PD). No significant separation was observed between groups at baseline (p>0.05). The y-axis is log-scaled. This plot serves as a comparison to the T1 timepoint data presented in the leading figures.


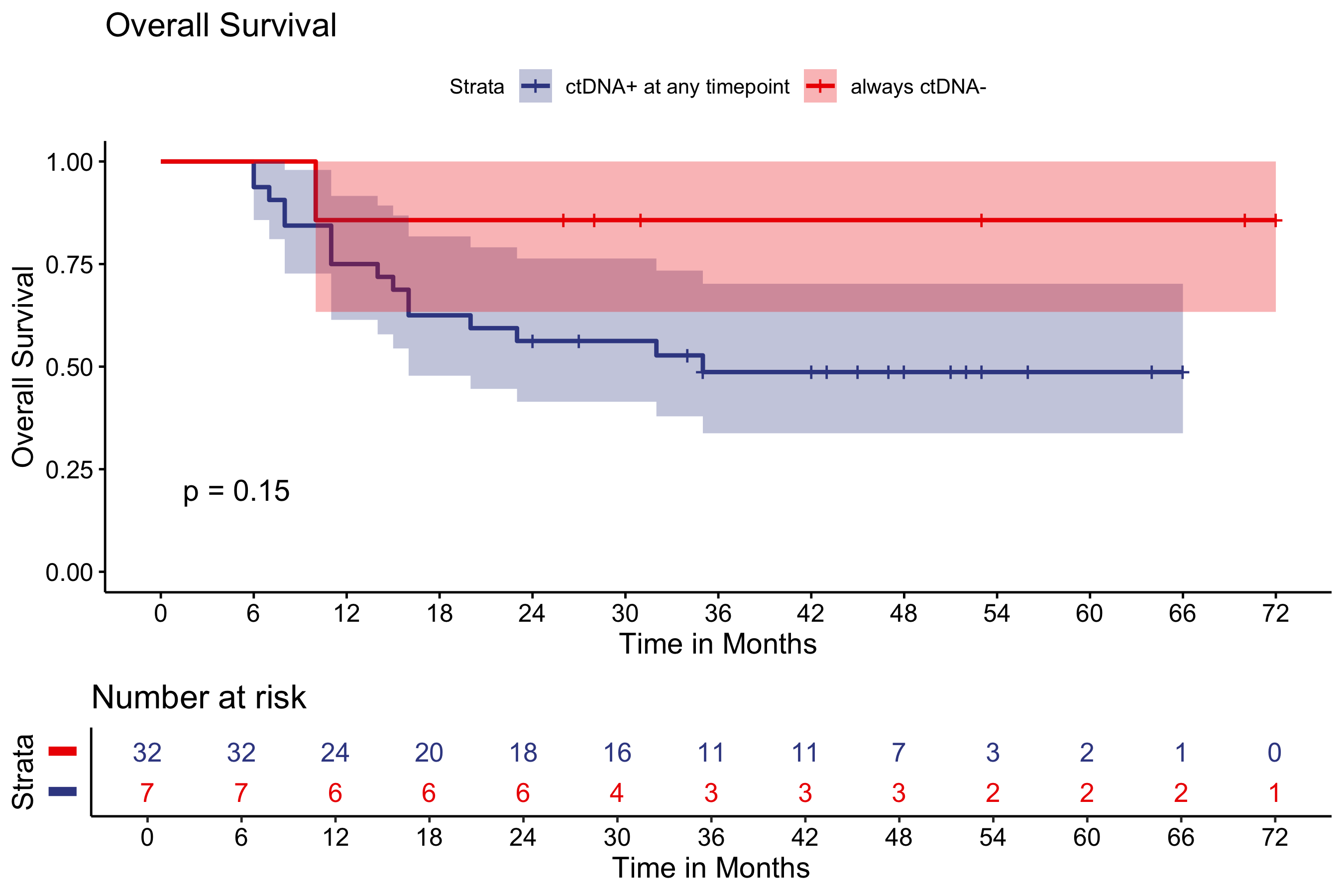


**Suppl. Figure 6:** Kaplan–Meier plot showing the overall survival (OS) probability of ctDNA-positive (at least one mutation detected) and ctDNA-negative patients at any timepoint during treatment (p=0.15)(N=39)


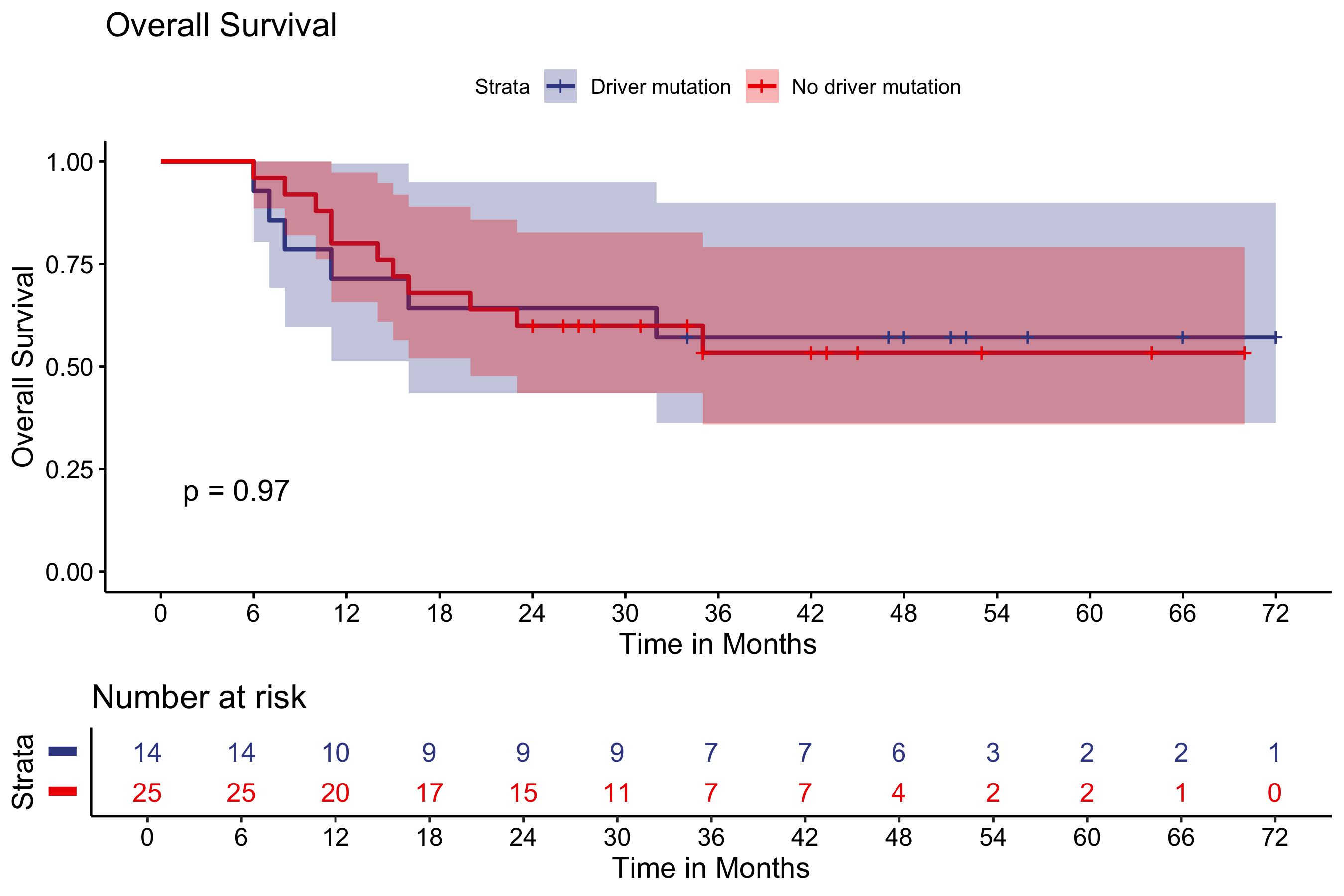


**Suppl. Figure 7:** Kaplan–Meier plot showing the overall survival (OS) probability of Driver-mutation versus non-driver-mutation during treatment (p=0.97), indicating unfavorable clinical outcomes in ctDNA-positive patients regardless of the variant. (N=39)
